# Molecular characterization of the 2022 Sudan virus disease outbreak in Uganda

**DOI:** 10.1128/jvi.00590-23

**Published:** 2023-09-26

**Authors:** Stephen Balinandi, Shannon Whitmer, Sophia Mulei, Charity Nassuna, Godfrey Pimundu, Tonny Muyigi, Markus Kainulainen, Elizabeth Shedroff, Inna Krapiunaya, Florine Scholte, Luke Nyakarahuka, Alex Tumusiime, Jackson Kyondo, Jimmy Baluku, Jocelyn Kiconco, Julie R. Harris, Alex R. Ario, Atek Kagirita, Henry K. Bosa, Isaac Ssewanyana, Susan Nabadda, Henry G. Mwebesa, Jane R. Aceng, Diana Atwine, Julius J. Lutwama, Trevor R. Shoemaker, Joel M. Montgomery, Pontiano Kaleebu, John D. Klena

**Affiliations:** 1 Uganda Virus Research Institute, Entebbe, Uganda; 2 Viral Special Pathogens Branch, Centers for Disease Control and Prevention, Atlanta, Georgia, USA; 3 Uganda National Health Laboratory Services, Ministry of Health, Kampala, Uganda; 4 College of Veterinary Medicine, Animal Resources and Biosecurity, Makerere University, Kampala, Uganda; 5 Uganda Public Health Fellowship Program, Kampala, Uganda; 6 Ministry of Health, Kampala, Uganda; 7 Kellogg College, University of Oxford, Oxford, United Kingdom; 8 MRC/UVRI & LSHTM Uganda Research Unit, Entebbe, Uganda; University of Kentucky College of Medicine, Lexington, Kentucky, USA

**Keywords:** Sudan virus, Uganda, viral hemorrhagic fever, Ebola virus

## Abstract

Uganda experienced five Ebola disease outbreaks caused by Bundibugyo virus (*n* = 1) and Sudan virus (SUDV) (*n* = 4) from 2000 to 2021. On 20 September 2022, Uganda declared a fifth Sudan virus disease outbreak in the Mubende district, resulting in 142 confirmed and 22 probable cases by the end of the outbreak declaration on 11 January 2023. The earliest identified cases, through retrospective case investigations, had onset in early August 2022. From the 142 confirmed cases, we performed unbiased (Illumina) and SUDV-amplicon-specific (Minion) high-throughput sequencing to obtain 120 SUDV genome-and coding-complete sequences, representing 95.4% (104/109) of SVD-confirmed individuals within a sequence-able range (Ct ≤30) and 10 genome sequences outside of this range and 6 duplicate genome sequences. A comparison of the nucleotide genetic relatedness for the newly emerged Mubende variant indicated that it was most closely related to the Nakisamata SUDV sequence from 2011, represented a likely new zoonotic spillover event, and exhibited an inter- and intra-outbreak substitution rate consistent with previous outbreaks. The most recent common ancestor for the Mubende variant was estimated to have occurred in October and November 2021. The Mubende variant glycoprotein amino acid sequences exhibited 99.7% similarity altogether and a maximum of 96.1% glycoprotein similarity compared to historical SUDV strains from 1976. Integrating the genetic sequence and epidemiological data into the response activities generated a broad overview of the outbreak, allowing for quick fact-checking of epidemiological connections between the identified patients.

**IMPORTANCE:**

Ebola disease (EBOD) is a public health threat with a high case fatality rate. Most EBOD outbreaks have occurred in remote locations, but the 2013–2016 Western Africa outbreak demonstrated how devastating EBOD can be when it reaches an urban population. Here, the 2022 Sudan virus disease (SVD) outbreak in Mubende District, Uganda, is summarized, and the genetic relatedness of the new variant is evaluated. The Mubende variant exhibited 96% amino acid similarity with historic SUDV sequences from the 1970s and a high degree of conservation throughout the outbreak, which was important for ongoing diagnostics and highly promising for future therapy development. Genetic differences between viruses identified during the Mubende SVD outbreak were linked with epidemiological data to better interpret viral spread and contact tracing chains. This methodology should be used to better integrate discrete epidemiological and sequence data for future viral outbreaks.

## INTRODUCTION

Ebola disease (EBOD) caused by the *Orthoebolavirus* genus (1) remains one of the most challenging public health threats for global communities in the modern era. When outbreaks occur, they trigger immense fear in the general population and devastate health and the economy (2 – 4). Patients with EBOD usually present with acute fever and progress rapidly into fulminant disease with multi-organ involvement and subsequent hemorrhagic manifestations (5). Death usually occurs in at least 50% of EBOD victims (6).

Ebola virus disease (EVD) caused by the *Orthoebolavirus zairense* species was first definitively described in 1976 when simultaneous outbreaks occurred in the villages of Yambuku (then in Zaire, now the Democratic Republic of the Congo) and Nzara (then in Sudan, now in South Sudan) (7 – 9). For the next 20 years, human cases were rare and geographically localized within the Middle Africa region (10). It is hypothesized to circulate in pteropodid bats and possibly other non-human primates that act as its natural reservoirs (11). However, outbreaks of EBOD have become more frequent, with almost 70% of the total outbreaks recorded after the year 2000 (10). The 2013–2016 Western Africa EVD outbreak, which involved >28,000 cases (10, 12), remains the largest on record. With rapidly increasing international traffic and trade amidst an increasing human population, deforestation, and rural urbanization, the pandemic potential for EBOD is increasing (13). As a result, there is increasing concern that its natural occurrence may be geographically wider than previously thought (14, 15).

Orthoebolaviruses, some of which cause EBOD, belong to the *Filoviridae* family, a group of enveloped, filamentous viruses with non-segmented, negative-sense RNA genomes of approximately 19 kb. Currently, four orthoebolaviruses are pathogenic to humans [Ebola virus (EBOV), Sudan virus (SUDV), Bundibugyo virus (BDBV), and Taï Forest virus], with no evidence of acute human disease attributed to the other two member species [Bombali virus and Reston virus (RESTV) (but anti-RESTV antibodies have been detected in a single individual)] (16, 17). Previous EBOD outbreaks in Uganda have been associated with both BDBV (Bundibugyo district, 2007) and SUDV (Gulu district, 2000; Luweero district, 2011 and 2012; and Kibaale district, 2012) (10, 18). In this paper, we report on the genomic characterization of the SUDV variant that caused the 2022 Sudan virus disease (SVD) outbreak, first identified in September 2022 after SVD was confirmed in a 26-year-old male from Maduddu sub-county, Mubende district (19). The value of genomic data to the advancement of filovirology was recently highlighted (20) and was found to be a useful tool during response to ongoing outbreaks (21, 22). It is also worth noting that the amount of genomic data for SUDV that are currently available in GenBank and other databases is limited, when compared to its EBOV counterpart, perhaps partly due to SUDV’s infrequent occurrence and relatively smaller outbreaks (23); thus, it presents a challenge to developing medical countermeasures.

## MATERIALS AND METHODS

### Patient enrollment and sample collection

In this investigation, participants were initially sampled when they met the established criteria for suspected viral hemorrhagic fevers (VHFs) in Uganda. Briefly, these suspected cases presented with either a clinical sign [acute onset of fever (>38°C)] and other clinical symptoms or were epidemiologically linked to a confirmed case. In all cases, whole blood samples were collected and submitted for EBOD confirmation to the VHF Laboratory at Uganda Virus Research Institute (UVRI) in Entebbe or Mubende Mobile Laboratory at Mubende Regional Referral Hospital, Mubende district.

### Nucleic acid extractions and RT-PCR

At UVRI, samples were processed for EBOD confirmation using previously reported methods (18, 24). Briefly, the MagMax Kit (Applied Biosystems Inc., Vilnius, Lithuania) was used to extract RNA, followed by RT-PCR on ABI’s QuantStudio 5 or 7500 Real-Time PCR System instruments (Applied Biosystems), using the US CDC custom primers and probes that target the NP region for SUDV [EboSudBMG 1(+) 5′-GCC ATG GIT TCA GGT TTG AG-3′, EboSudBMG 1(−) 5′-GGT IAC ATT GGG CAA CAA TTC A, and EboSudBMG Probe 5′FAM-AC GGT GCA CAT TCT CCT TTT CTC GGA-BHQ1] (25). While at the Mubende Field Laboratory, SVD was confirmed using the RealStar Filovirus Kits (Altona Diagnostics, Hamburg, Germany), according to the manufacturer’s instructions (26). However, all samples confirmed at Mubende were transferred to UVRI, where a repeat test using the CDC protocol (as stated above) was done.

### High-throughput sequencing and bioinformatics

High-throughput sequencing was performed using either unbiased library preparation and Illumina sequencing or SUDV-amplicon-specific library preparation and minion-based sequencing. Samples with a cycle threshold (Ct) ≤25 were prepared for Illumina sequencing by treating with RNase-free DNase (Roche, Basel, Switzerland), and depleting host rRNA with the NEBNext rRNA Depletion Kit v2, followed by library preparation using the NEBNext Ultra II Directional RNA Library Preparation Kit (New England Biolabs, Beverly, MA). Libraries were then sequenced using either an Illumina iSeq100 (V1 2 × 150 cycles) or a MiSeq (2 × 150 cycles). In the beginning of the outbreak, all samples were sequenced using unbiased library preparation (Illumina) and after an SUDV consensus genome sequence was generated, the amplicon-based minion protocol was developed and deployed (after an initial validation where duplicate samples were sequenced using both Illumina and Minion methods). Samples with 25 < Ct < 30, and another subset of samples with Ct ≤25, were sequenced using SUDV-specific primers (Table S1) and the ARTIC protocol (https://www.protocols.io/view/ebola-virus-sequencing-protocol-e6nvw9p7dgmk/v1) (27, 28).

Consensus genome sequences were constructed using the bioinformatics method appropriate to the library construction method—either the ARTIC EBOV bioinformatics protocol (for amplicon-based MinIon sequencing) or a read mapping to a reference genome sequence using in-house scripts (https://github.com/evk3/ UVRI_Sudan_EBOV_Uganda_2022 for TruSeq-based Illumina sequencing). Configuration files were changed to trim SUDV primer pools from the reads for the artic EBOV bioinformatics protocol. For TruSeq-based Illumina sequencing, low-quality reads/bases were filtered using Prinseq-lite v0.20.3 (-min_qual_mean 25 -trim_qual_right 20 -min_len 50), and SUDV genome sequences were assembled by aligning trimmed reads to the Nakisamata 2011 outbreak sequence (JN638998) using BWA-mem (29) and iteratively mapped to the intermediate scaffold genome sequence; new consensus genome sequences were called using samtools mpileup (-A -aa -d 6000000 -B -Q 0) and ivar consensus (1.3.1) (-m 2 -n N). Genome sequences were deposited into GenBank with accession numbers OQ672950–OQ673069.

### Tempest, Bayesian analysis, and phylogeographic reconstruction

Root vs tip date divergence was performed using TempEst (v1.5.1) (30) to estimate the clock-like nature of inter- and intra- outbreak substitution rates. SUDV alignments were made using MAFFT (v7.450) (31), while its maximum likelihood trees were made using RAxML (v7.3.0) (32). Trees were rooted to the earliest available inter- and intra-outbreak sequences, MK952150 Maridi or OQ672950 2022002242_C008, respectively. Since the 2022002242_C008 sequence was collected nearly 2 months after the beginning of the Mubende outbreak, the intra-outbreak root age was estimated using the correlation model in TempEst (30). A single sample sequence missing the collection and symptom onset dates (2022005178) was removed from the intra-outbreak rate analysis.

All Bayesian analysis was performed using BEAST (v1.10.4) (33). SUDV alignments were split into coding and non-coding regions with unlinked substitution models. Model parameter testing was performed using the GTR+Γ (4) nucleotide substitution model, different clocks (fixed local clock, strict or uncorrelated relaxed lognormal clocks set with an initial prior value of 1 × 10^−3^ subs/site/year), and tree parameters [constant, exponential, or Skygrid (inter-outbreak: time at last transition point = 47.0 and 94 grid points; intra-outbreak: time at last transition point = 47.0 and 564 (or 94) grid points)]; the new tree operator mix and strength of model fit were assessed using Bayes factors calculated from path sampling and stepping stone analysis (Table S2). Each analysis was run for 4 (inter-outbreak) or 3 (intra-outbreak) independent replicates consisting of 100M states logged every 10,000th state, and 10% burn-in was removed from each replicate (in 3/16 replicates with a higher burn-in of 17.5%, 23%, or 40% was removed before convergence was met). Joint and prior ESS values were >200 for all models.

Phylogeographic reconstruction was performed using Nextstrain (v5.0.1) and visualized using Python (v3.9.13) with the Baltic and matplotlib packages.

### Nucleotide and amino acid comparisons

Nucleotide and amino acid entropy were calculated using Nextstrain with Auspice (v2.43.0) (34). Ancestral sequences were estimated for the root and internal nodes, while entropy was calculated as a count of the observed and inferred mutations relative to the total number of sequences in the tree. Glycoprotein full-length amino acid alignments were generated using MAFFT (v7.490) (31) in Geneious Prime (2022.1.1) (www.geneious.com/) for all available historic SUDV sequences and from Mubende outbreak sequences. The protein similarity was determined using BLOSUM62 (35), while percentage similarity was calculated using the BLOWSUM62 with threshold = 1 from Geneious Prime. When comparing multiple sequences, we selected the lowest percent similarity score.

## RESULTS

**Fig 1:**
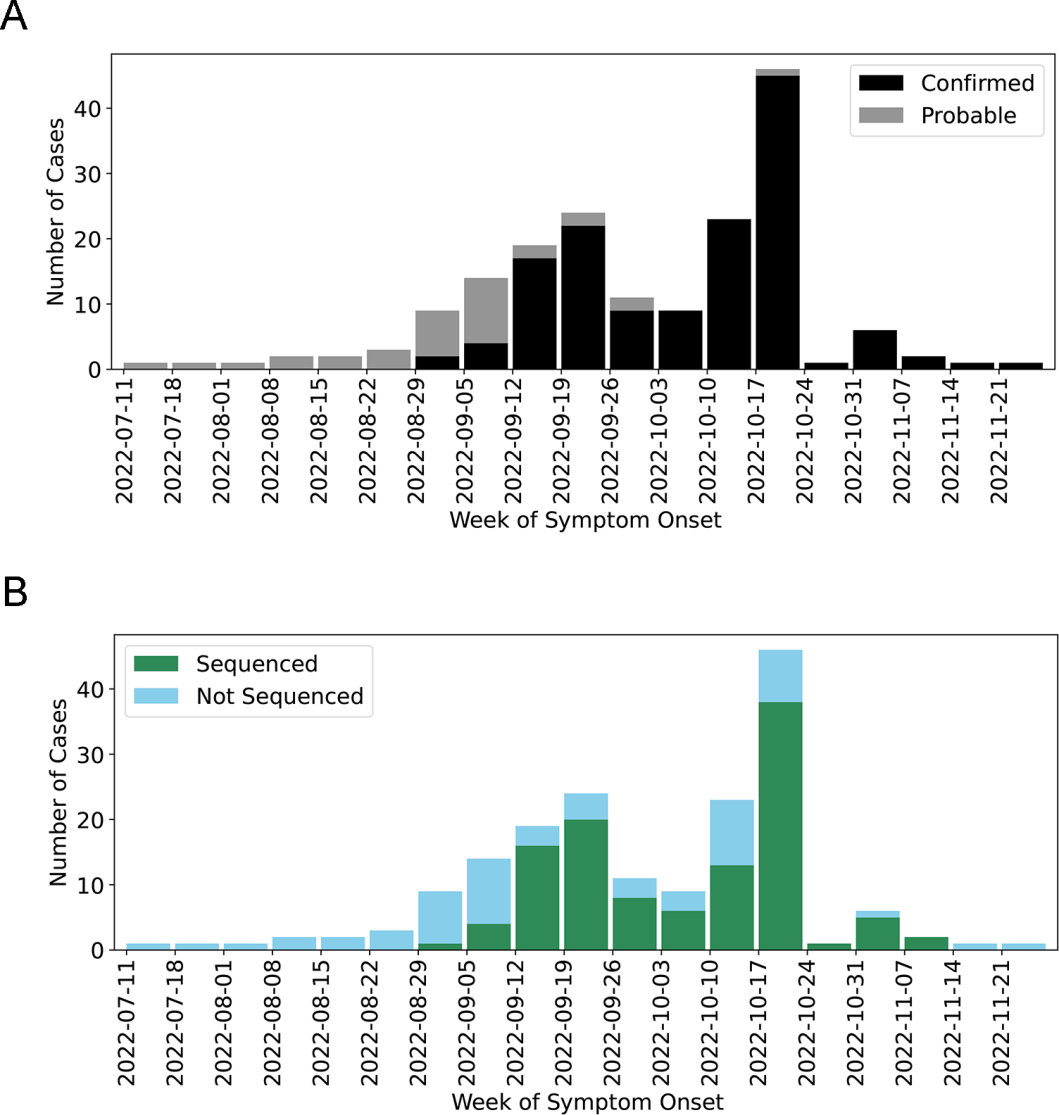
Epidemiological curve and the ratio of sequenced to total samples over time. (**A**) Epidemiological curve including probable and confirmed cases. (**B**) The ratio of sequenced to total samples over time.

On 19 September 2022, SVD was identified in an individual from Mubende District, Uganda, by the VHF Laboratory at UVRI. On 20 September 2022, the Ministry of Health of Uganda officially declared an SVD outbreak. There were 142 confirmed and 22 probable SVD cases with a case fatality rate of 36.6% (52/142) among confirmed cases, and the outbreak was declared over on 11 January 2023 (42 days, or two incubation periods after the last case). During the outbreak, UVRI attempted sequencing on 129 specimens and generated 120 genome sequences with greater than 90% coverage from 114 unique cases (Fig. 1). Viruses from six individuals were sequenced twice from two specimens collected on different dates. Specimens were not available from all the probable cases. Therefore, the sequence data presented here represent 95.4% (104/109) of SVD-confirmed individuals that contain SUDV RNA within a predetermined (data not shown) sequence-able range (Ct <30) and eight genome sequences from SVD-confirmed individuals outside of this range.

**Fig 2:**
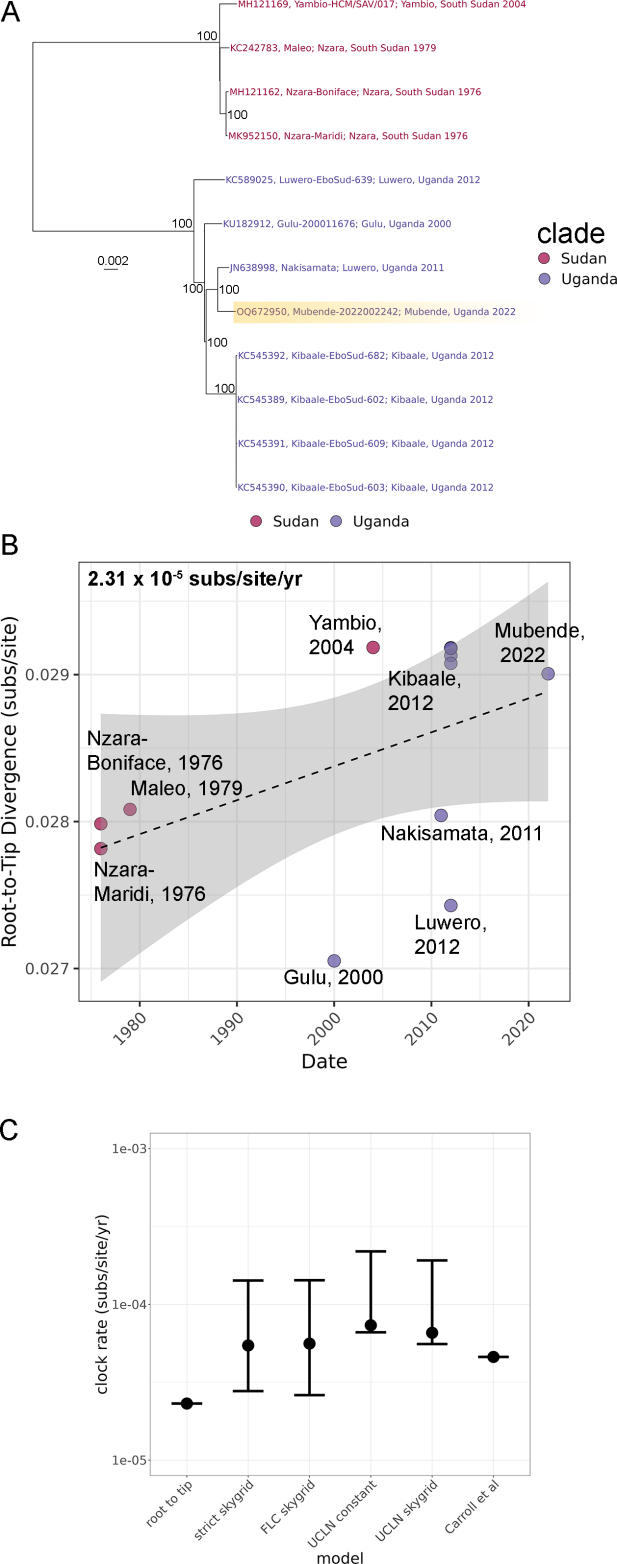
*Orthoebolavirus sudanense* species inter-outbreak inferred evolutionary relationships. (**A**) Maximum likelihood phylogenetic tree for all available full-length SUDV sequences. The tree is midpoint rooted, and the outbreak locations (Sudan vs Uganda) are indicated by color. Bootstrap support values (gray) greater than 70% are indicated at nodes (*n* = 1,000 replicates). (**B**) Divergence from root vs time demonstrates the clock-like nature of the *Orthoebolavirus sudanense* species substitution rate (dotted line). The confidence interval is shaded gray. (**C**) Substitution rate estimates compared across different Bayesian models, the root-to-tip analysis from (B), and the historic analysis from Carroll et al.

The earliest available sequence, from an individual with symptom onset of 11 September 2022 (approximately 2 months after the estimated start of the outbreak), represented a likely new SUDV spillover and was most closely related to the May 2011 Nakisamata outbreak sequence from Luwero District, Uganda (36), instead of the more recent SUDV genome sequences from the SVD outbreak in Kibaale (located approximately 60 km from Mubende) in 2012 (Fig. 2A; Fig. S1) (18). The May 2011 Nakisamata outbreak sequence and 2022 Mubende variant (2022002242 isolate) are 99.58% identical (86 base pair differences) and only differed by 10 amino acids.

The inter-outbreak mutation rate, including the new Mubende variant (2022002242 isolate) sequence, was clock-like (Fig. 2B) and similar among different Bayesian models (Fig. 2C). The best-fit model (UCLN, Skygrid) inter-outbreak rate was 6.5705 × 10^−5^ subs/site/year (1.0050 × 10^−5^–1.2584 × 10^−4^, 95% highest posterior density (HPD) estimates), similar to the SUDV inter-outbreak rate of 4.6 × 10^−5^ subs/site/year (Fig. 2B) previously observed by Carroll et al. (37).

**Fig 3:**
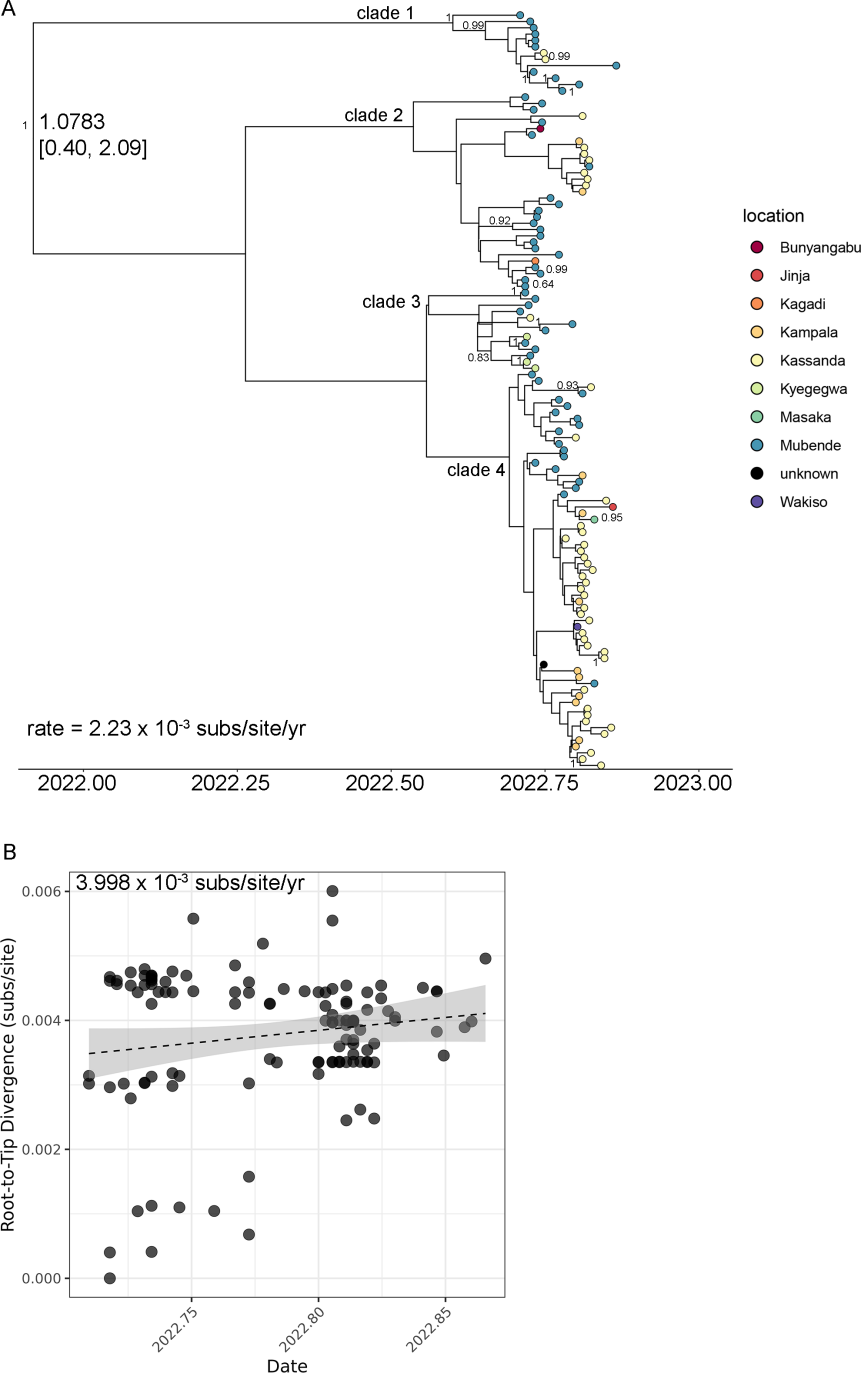
Sudan virus, Mubende variant intra-outbreak inferred evolutionary relationships. (**A**) Time-scaled phylogeny with leaves colored according to residence district. Nodes with posterior support greater than 0.6 are labeled in red. Node age estimates with 95% HPD intervals are at selected internal nodes (black). (**B**) Divergence from root vs time demonstrates the clock-like nature of the Mubende variant substitution rate (dotted line). The confidence interval is shaded gray. Root age was estimated at 1 November 2021.

The phylogenetic tree formed four distinct clades, although the overall tree topology was poorly supported (Fig. 3). Over time, the outbreak spread from Mubende District to other geographic areas, including the neighboring Kassanda District and Kampala Metropolitan Area (Fig. S2). The sequences from the Mubende outbreak exhibited a clock-like rate (Fig. 3B). They demonstrated an intra-outbreak rate of 2.23 × 10^−3^ (1.274–3.179 × 10^−3^ subs/site/year) or 3.998 × 10^−3^ subs/site/year using a Bayesian and correlative approach, respectively (Fig. 3A and B). The Bayesian method estimated MRCA for the Mubende SUDV clade was 1.0783 years (15 October 2021) [0.4068 (17 June 2022)–2.0935 (9 October 2020), 95% HPD interval] before the most recent case, and the split from the most closely related 2011 Nakisamata outbreak sequence to have occurred 11.7442 years (14 February 2011) [11.5453 (27 April 2011)–12.0357 (30 October 2010), 95% HPD interval] before the most recent case (Fig. 3A). The correlated approach (using Tempest) also estimated the age of the Mubende SUDV MRCA to be 1.0301 years (2 November 2021).

**Fig 4:**
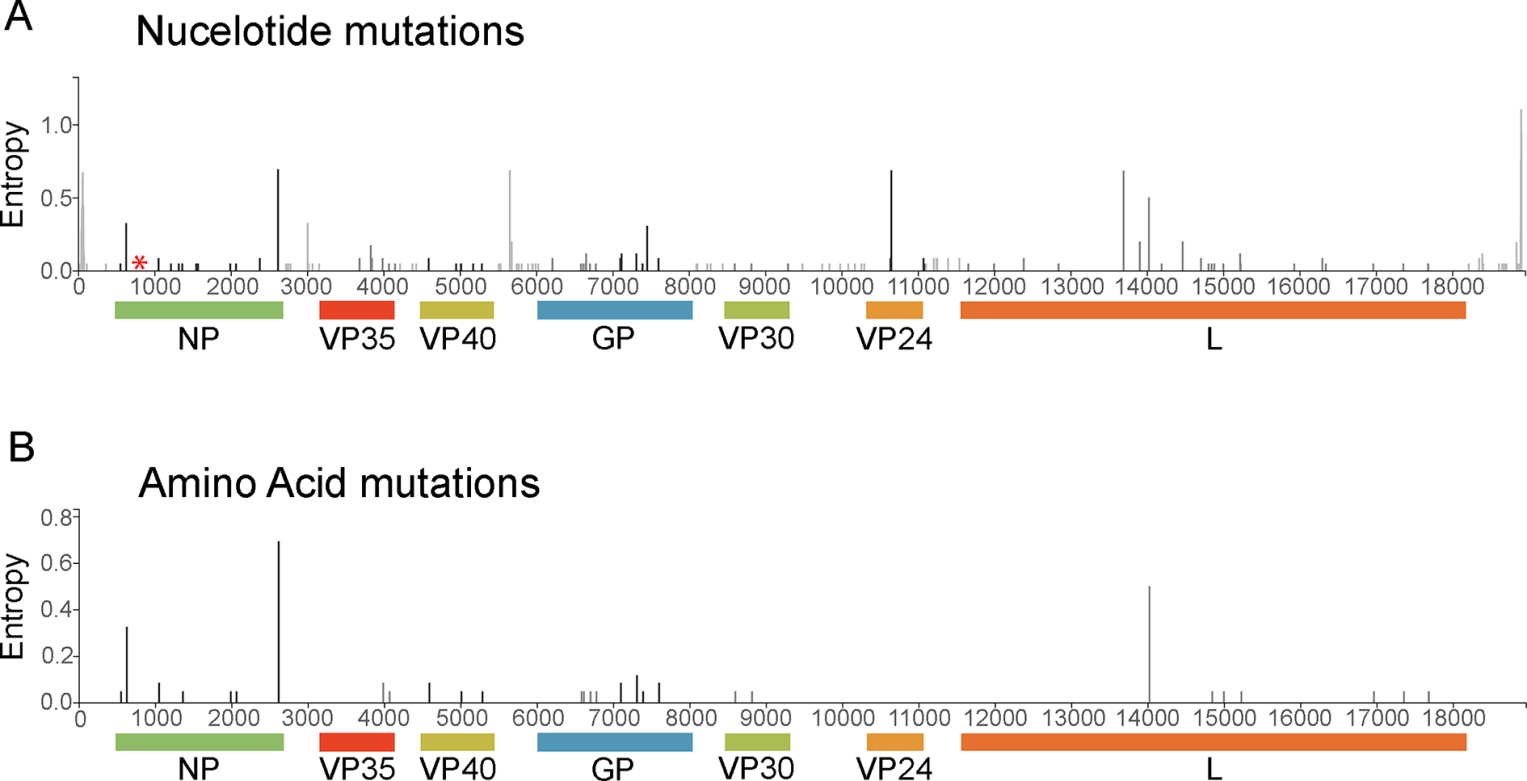
The prevalence of nucleotide and amino acid mutations during the SUDV, Mubende variant outbreak. Mutations are relative to the inferred root ancestral sequence, and entropy is a count of the observed and inferred mutations relative to the total number of sequences in the tree. (**A**) Entropy of nucleotide mutations from Mubende variant sequences (*n* = 120). A red asterisk indicates the location of the UVRI SUDV qRT-PCR diagnostic assay primer and probe binding sites. (**B**) Entropy of non-silent amino acid mutations from Mubende variant sequences (*n* = 120).

The prevalence of nucleotide and amino acid mutations during the outbreak was also assessed (Fig. 4). Most nucleotide mutations were silent (Fig. 4A), and a minority of amino acid mutations reached prevalence during the outbreak (Fig. 4B). The most prevalent mutations occurred in the NP (codon 711) and polymerase (codon 821) regions relative to the inferred root ancestral sequence (online supplementary file 1). These mutations did not interfere with the diagnostic assays used at UVRI, and it is currently unknown whether the mutations impacted the performance of the commercially available RealStar Filovirus Screen RT-PCR Kit 1.0 and RealStar Filovirus Type RT-PCR Kit 2.0 assays run in the Mubende mobile laboratory (26) since the primer and probe sequences are proprietary. The similarity between the known SUDV glycoprotein (GP) sequences was also assessed since the cAd3-EBO vaccine expresses the SUDV glycoprotein (38). The Mubende glycoprotein amino acid sequences exhibited 99.7% similarity altogether and a maximum of 96.1% similarity compared to historical SUDV sequences from 1976 (Boniface and Maridi) (Table 1). Most of the amino acid differences between the historical and current SUDV GPs occurred in the glycan cap and mucin-like domains; most of the Mubende sequences were highly similar and differed only in 1–2 amino acids.

**TABLE 1:** SUDV glycoprotein similarity^*a*^

	1976 Nzara-Boniface	1976 Nzara-Maridi	1979 Maleo	2000 Gulu-200011676	2004 Yambio- HCM/SAV/017	2012 Kibaale	2012 Luwero-Ebo639	2011 Nakisamata	Mubende variant, all sequences
1976 Nzara-Boniface, MH121162									
1976 Nzara-Maridi, MK952150	100								
1979 Maleo, KC242783	99.8	99.8							
2000 Gulu-200011676, KU182912	96.4	96.4	96.5						
2004 Yambio-HCM/SAV/017, MH121169	99.7	99.7	99.8	96.4					
2012 Kibaale, KC545389-91	96.3	96.3	96.4	99.7	96.3				
2012 Luwero-Ebo639, KC589025	96.4	96.4	96.5	99.4	96.4	99.1			
2011 Nakisamata, JN638998	96.4	96.4	96.5	100	96.4	99.7	99.4		
Mubende variant, all sequences	96.1	96.1	96.3	99.7	96.1	99.4	99.1	99.7	99.7

^*a*^ We compared the GP amino acid sequences between historic SUDV sequences and sequences from the Mubende outbreak (*n* = 112). Eight strains with incomplete GP coverage were removed from the analysis. When comparing multiple sequences (e.g., Kibaale, Mubende), we selected the lowest similarity score. The Mubende vs Mubende comparison represents the amount of intra-outbreak GP similarity.

**Fig 5:**
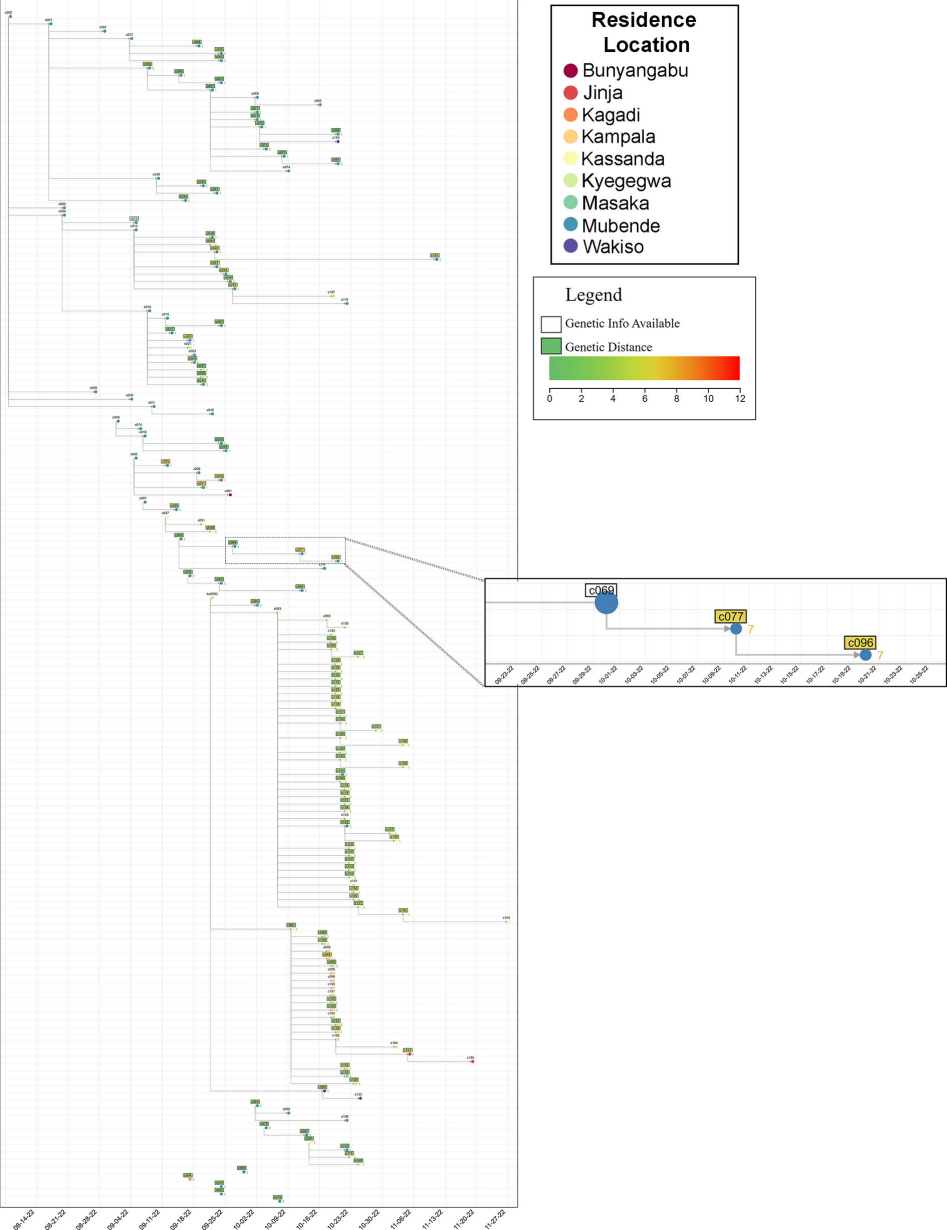
Combining epidemiological and phylogenetic networks provides a wide-scale view of the Mubende SUDV outbreak. Circular nodes represent single individuals, are located a symptom onset dates (*x*-axis), and are colored according to residence district. Shading in boxes (green to orange) indicates genetic distance relative to the earliest sequence in the outbreak. Inset demonstrates a contact tracing chain consisting of three individuals. Numbers next to nodes indicate the number of raw nucleotide differences relative to the C069 sequence.

Using epidemiological contact-tracing data, an extensive view of the SVD outbreak in the Mubende district was constructed, including genetic data (when available), which allowed the geographic and temporal spread of the outbreak to be visualized (Fig. 5). The ChainChecker tool allowed for fact-checking of epidemiological connections based on genetic data (39), and the epidemiologically reported geographic spread was consistent with the phylogenetically reconstructed spread (Fig. S2). For example, as shown in Fig. 5, case C069 was epidemiologically linked to case C077. However, the genetic data indicated that C069 was not closely related to C077, and the number of genetic differences (*n* = 7) was higher than what the substitution rate would predict for this exposure window [10 days, expect 1.2 (0.7–1.6) differences], suggesting that C077 was infected by a source other than C069.

## DISCUSSION

Here, a new SUDV variant that caused an SVD outbreak in Mubende, Uganda, from early August to November 2022, is characterized. The Mubende variant was likely a single zoonotic spillover from an unknown reservoir; the inter-outbreak substitution rate is consistent with this spillover scenario and previous inter-outbreak rate estimates (37). Furthermore, the Mubende variant likely emerged in October and November 2021, and the SUDV acute substitution rate estimates are consistent with previous EBOV estimates (2.23 × 10^−3^ subs/site/year vs ~1 × 10^−3^ sub/site/year) (40 – 43). Finally, the Mubende variant did not exhibit any mutations in the SUDV diagnostic assay binding site, and only minor amino acid mutations were found in viral proteins. However, the phenotype of the Mubende variant needs to be further investigated, as previously implied for EBOV (44). Nevertheless, given that these mutations only differed a maximum of 3.9% from other SUDV sequences and occurred mainly in the glycan cap and mucin-like domains, we believe that vaccine-derived glycoprotein antibodies are likely to cross-react with the Mubende variant and that SUDV GP-containing vaccines could reduce viral spread during future SVD outbreaks.

During the Mubende SVD outbreak, the UVRI laboratory performed real-time sequencing and quickly shared these data with the outbreak response command structure. The sequence data were combined with the epidemiological data to create a wider view of the outbreak using the ChainChecker application (39). Over time, the sequence data supported the contact tracing chains, and in some instances, the epidemiological connections were re-evaluated due to the genetic data, described in further detail by Kabami et al. (45). Furthermore, when SVD expanded outside of the Mubende district, UVRI prioritized the sequencing of specific samples to better understand the contact tracing chains. Unfortunately, not all samples from the outbreak could be sequenced due to lower viral loads (*n* = 28) at the time of case detection. In limited instances (*n* = 3), a connection between the sequenced specimen and a confirmed case could not be made. Based on the epidemiological data, four chains remained unconnected to the extensive contact tracing network. Using genetic data, however, we can now hypothetically link these chains to the more extensive contact tracing network.

### Conclusions

This work demonstrates that the *Orthoebolavirus sudanense* species continues to evolve slower than the *Orthoebolavirus zaireense* species as previously established (37). These data are, therefore, encouraging and suggestive that future *Orthoebolavirus sudanense* species will be susceptible to vaccine-derived cross-reacting SUDV antibodies. Furthermore, by integrating genetic and epidemiological data, a broad view of the SVD outbreak was generated, allowing for the fact-checking of epidemiological connections and pre-existing assumptions.

## SUPPLEMENTAL MATERIAL

Legends of supplemental materials jvi.00590-23-s0001.docx Titles of all supplemental figures and tables.

Supplemental Figure S1 jvi.00590-23-s0002.tif Orthoebolavirus sudanense species inter-outbreak inferred evolutionary relationships.

Supplemental Figure S2 jvi.00590-23-s0003.tif Phylogeographic reconstruction of the Mubende outbreak. Time-scaled phylogeny for all available full-length Mubende outbreak sequences. Branch color indicates the inferred geographic spread during the outbreak. Leaf color represents residence district for individuals.

Supplemental file 1 jvi.00590-23-s0004.pdf Inferred root ancestral sequence from Nextstrain. It is related to Figure 4.

Supplemental Table S1 jvi.00590-23-s0005.xlsx SUDV amplicon primers for minion sequencing.

Supplemental Table S2 jvi.00590-23-s0006.xlsx Strength of Bayesian model fit.

ASM does not own the copyrights to Supplemental Material that may be linked to, or accessed through, an article. The authors have granted ASM a non-exclusive, world-wide license to publish the Supplemental Material files. Please contact the corresponding author directly for reuse.
