# Supplementary material for: Molecular characterization of the 2022 Sudan virus disease outbreak in Uganda
Source: J Virol. 2023 Sep 26;97(10):e00590-23. doi: 10.1128/jvi.00590-23 (PMC10617429; doi:10.1128/jvi.00590-23)
Supplement: Legends of supplemental materials — Titles of all supplemental figures and tables. [file jvi.00590-23-s0001.docx]

**Supplemental Legends**

**Supplemental Table 1:** SUDV amplicon primers for minion sequencing

**Supplemental Table 2:** Strength of Bayesian Model Fit.

**Supplemental File 1:** Inferred root ancestral sequence from Nextstrain. It is related to Figure 4.

**Supplemental Figure 1:** *Orthoebolavirus sudanense* species inter-outbreak inferred evolutionary relationships. Maximum likelihood phylogenetic tree for all available full-length SUDV sequences. Tree is midpoint rooted, and the outbreak locations (Sudan, historic Uganda and Mubende) are indicated by color. Bootstrap support values (grey) greater than 70% are indicated at nodes (n=1000 replicates).

**Supplemental Figure 2:** Phylogeographic reconstruction of the Mubende outbreak. Time-scaled phylogeny for all available full-length Mubende outbreak sequences. Branch color indicates the inferred geographic spread during the outbreak. Leaf color represents residence district for individuals.
