# Supplementary figures and images for: Molecular characterization of the 2022 Sudan virus disease outbreak in Uganda
Source: J Virol. 2023 Sep 26;97(10):e00590-23. doi: 10.1128/jvi.00590-23 (PMC10617429; doi:10.1128/jvi.00590-23)

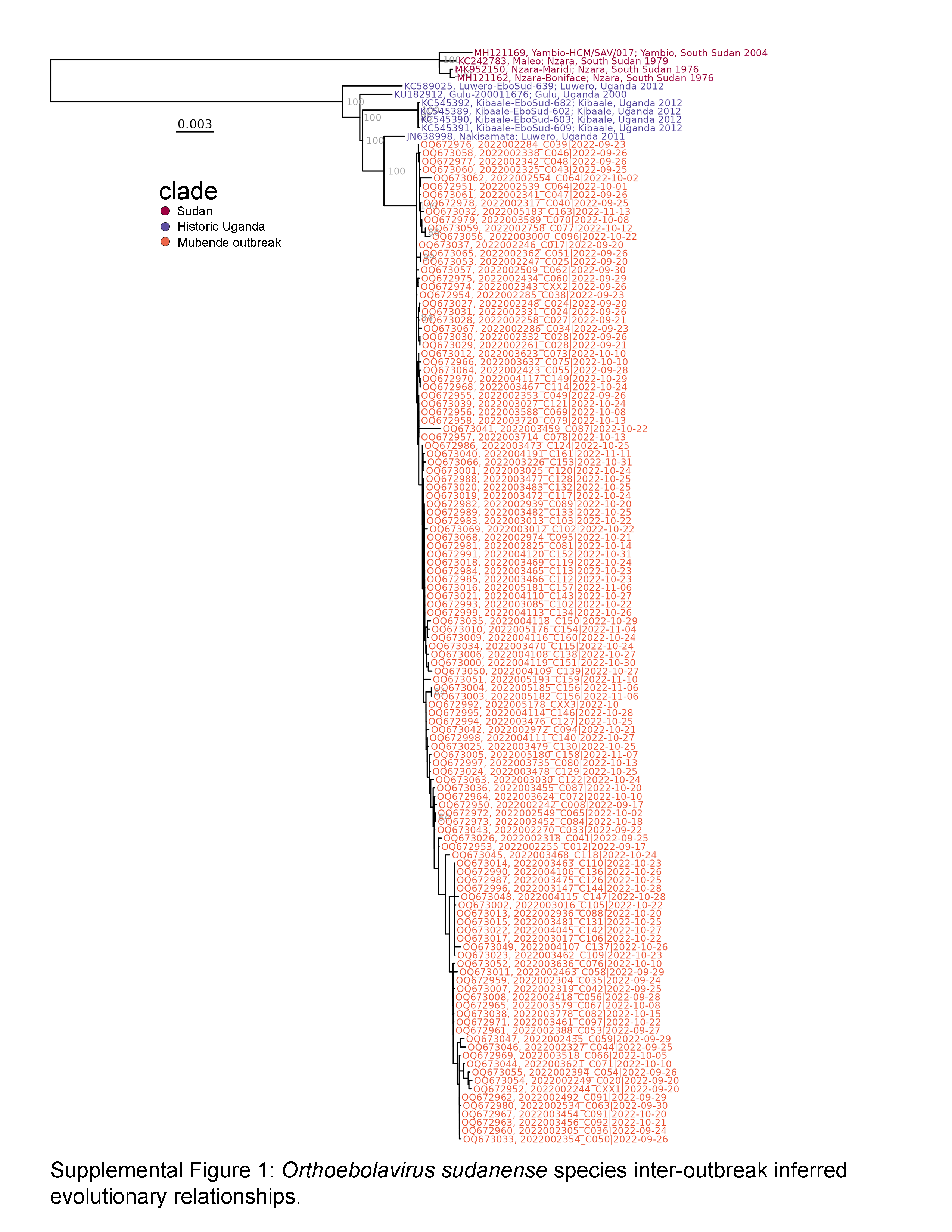

Supplement: Supplemental Figure S1 — Orthoebolavirus sudanense species inter-outbreak inferred evolutionary relationships. [file jvi.00590-23-s0002.tif]

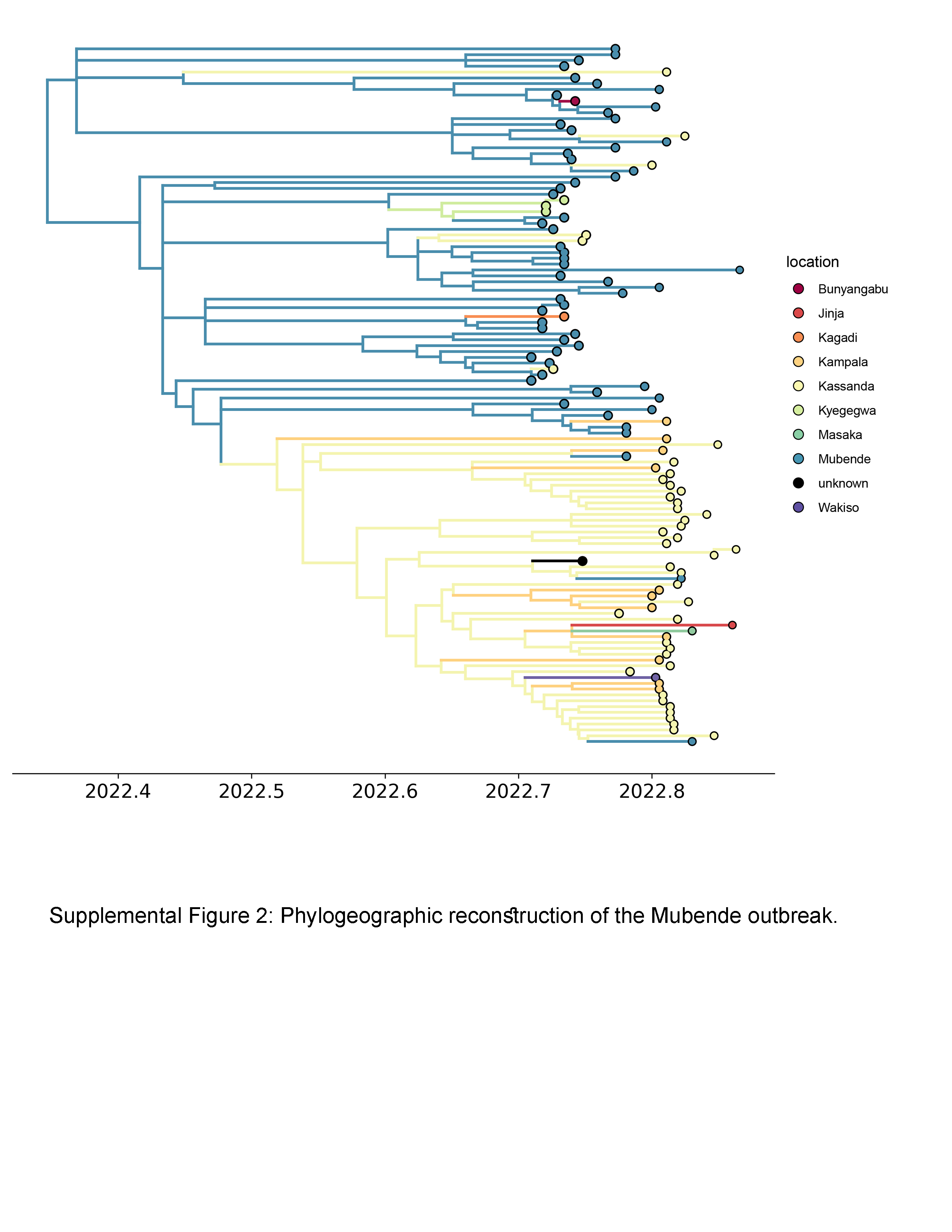

Supplement: Supplemental Figure S2 — Phylogeographic reconstruction of the Mubende outbreak. Time-scaled phylogeny for all available full-length Mubende outbreak sequences. Branch color indicates the inferred geographic spread during the outbreak. Leaf color represents residence district for individuals. [file jvi.00590-23-s0003.tif]
