# Supplementary material for: Molecular characterization of the 2022 Sudan virus disease outbreak in Uganda
Source: J Virol. 2023 Sep 26;97(10):e00590-23. doi: 10.1128/jvi.00590-23 (PMC10617429; doi:10.1128/jvi.00590-23)
Supplement: Supplemental file 1 — Inferred root ancestral sequence from Nextstrain. It is related to Figure 4. [file jvi.00590-23-s0004.pdf]

**GP_COOH**	**NLSEQLRGEELSFEALSLNETEDDDAASSRITKGRISDRATRKYSDLVPKNSPGMVPLHIPEGETTLPSQNSTEGR<br>RVGVNTQETITETAATIIGTNGNHMQISTIGIRPSSNQIPSSSPTTAPSPEAQTPTTHTSGPSVMATEEPTTPPGSS<br>PGPTTEAPTLTTPENITTAVKTVLPQESTSNGLITSTVTGILGSLGLRKRSRRQTNTKATGKCNPNLHYWTAQEQH<br>NAAGIAWIPYFGPGAEGIYTEGLMHNQNALVCGLRQLANETTQALQLFLRATTELRTYTILNRKAIDFLLRRWGGTC<br>RILGPDCCIEPHDWTKNITDKINQIIHDFIDNPLPNQDNDDNWWTGWRQWIPAGIGITGIIIAIIALLCVCKLLC***
**GP_NH3**	**MGSLSLLQLPRDKFRKSSFFVWVIILFQKAFSMPLGVVTNSTLEVTEIDQLVCKDHLASTDQLKSVGLNLEGSGVS<br>TDIPSATKRWGFRSGVPPKVVSYEAGEWAENCYNLEIKKPDGSECLPPPPDGVRGFPRCRYVHKAQGTGPCPGD<br>YAFHKDGAFFLYDRLASTVIYRGVNFAEGVIAFLILAKPKETFLQSPPIREAVNYTENTSSYYATSYLEYEIENFGAQ<br>HSTTLFKIDNNTFVRLDRPHTPQFLFQLNDTIHLHQQLSNTTGRLIWTLDANINADIGEWAFWENKK**
**L**	**MMATQHTQYPDARLSSPIVLDQCDLVTRACGLYSEYSLNPKLRTCRLPKHIYRLKYDTIVLRFISDVPVATIPIDYIA<br>PMLINVLADSKNVPLEPPCLSFLDEIVNYTVQDAAFLNYYMNQIKTQEGVITDQLKQNIRRVIHKNRYLSALFFWH<br>DLAILTRRGRMNRGNVRSTWFVTNEVVDILGYGDYIFWKIPIALLPMNTANVPHASTDWYQPNIFKEAIQGHTHII<br>SVSTAEVLIMCKDLVTSRFNTLLIAELARLEDPVSADYPLVDNIQSLYNAGDYLLSILGSEGYKIIKYLEPLCLAKIQLC<br>SQYTERKGRFLTQMHLAVIQTLRELLLNRGLKKSQLSKIREFHQLLLRLRSTPQQLCELFSIQKHWGHPVLHSEKAI<br>QKVKNHATVLKALRPIIIFETYCVFKYSVAKHFFDSQGTWYSVISDRCLTPGLNSYIRRNQFPPLPMIKDLLWEFYH<br>LDHPPLFSTKIISDLSIFIKDRATAVEQTCWDAVFEPNVLGYSPPYRFNTKRVPEQFLEQEDFSIESVLQYAQELRYL<br>LPQNRNFSFSLKEKELNVGRTFGKLPYLTRNVQTLCEALLADGLAKAFPSNMMVVTEREQKESLLHQASWHHTS<br>DDFGEHATVRGSSFVTDLEKYNLAFRYEFTAPFINYCNQCYGVRNVFDWMHFLIPQCYMHVSDYYNPPHNVTLE<br>NREYPPEGPSAYRGHLGGIEGLQQKLWTSISCAQISLVEIKTGFKLRSAVMGDNQCITVLSVFPLESSPNEQERCA<br>EDNAARVAASLAKVTSACGIFLKPDETFVHSGFIYFGKKQYLNGIQLPQSLKTAARMAPLSDAIFDDLQGTLASIGT<br>AFERSISETRHILPCRVAAAFHTYFSVRILQHHHLGFHKGSDLGQLAINKPLDFGTIALSLAVPQVLGGLSFLNPEKC<br>LYRNLGDPVTSGLFQLKHYLSMVGMSDIFHALVAKSPGNCSAIDFVLNPGGLNVPGSQDLTSFLRQIVRRSITLSA<br>RNKLINTLFHASADLEDELVCKWLLSSTPVMSRFAADIFSRTPSGKRLQILGYLEGTRTLLASKMISNNAETPILERL<br>RKITLQRWNLWFSYLDHCDPALMEAIQPIKCTVDIAQILREYSWAHILDGRQLIGATLPCIPEQFQTTWLKPYEQC<br>VECSSTNNSSPYVSVALKRNVVSAWPDASRLGWTIGDGIPYIGSRTEDKIGQPAIKPRCPSAALREAIELTSRLTWV<br>TQGSANSDQLIRPFLEARVNLSVQEILQMTPSHYSGNIVHRYNDQYSPHSFMANRMSNTATRLMVSTNTLGEFS<br>GGGQAARDSNIIFQNVINFAVALYDIRFRNTCTSSIQYHRAHIHLTNCCTREVPAQYLTYTTTLNLDLSKYRNNELIYD<br>SEPLRGGLNCNLSIDSPLMKGPRLNIIEDDLIRLPHLSGWELAKTVLQSIISDSSNSSTDPISSGETRSFTTHFLTYP<br>KIGLLYSFGALISFYLGNTILCTKKIGLTEFLYYLQNQIHNLSHRSLRIFKPTFRHSSVMSRLMDIDPNFSIYIGGTAG<br>DRGLSDAARLFLRIAISTFLSFVEEWVIFRKANIPLWVIYPLEGQRPDPPGEFLNRVKSLIIGTEDDKNKGSILSRSG<br>EKCSSNLVYNCKSTASNFFHASLAYWRGRHRPKKTIGATNATTAPHIILPLGNSDRPPGLDLNRNNDTFIPTRIKQI<br>VQGDSRNDRTTTTRFPPKSRSTPTSATEPPTKMYEGSTTHQGKLTDTHLDEDHNAKEFPSNPHRLVVPFFKLTKD<br>GEYSIEPSPEESRSNIKGLLQHLRTMVDTTIYCRFTGIVSSMHYKLDEVLWEYDKFESAVTLAEGEGSGALLLIQKY<br>GVKKLFLNTLATEHSIESEVISGYTTPRMLLSIMPKTHRGELEVILNNSASQITDITHRDWFSNQRNRIPTDADIITM<br>DAETTENLDRSRLYEAVYTIICNHINPKTLKVVILKVFLSDLDGMCWINNYLAPMFGSGYLIKPITSSAKSSEWYLCL<br>SNLLSTLRTTQHQTQANCLHVVQCALQQQVQRGSYWLSHLTKYTTSRLHNSYIAFGFPSLEKVLYHRYNLVDSRN<br>GPLVSITRHLALLQTEIRELVTDYNQLRQSRTQTYHFIKTSKGRITKLVNDYLRFELVIRALKNNSTWHHELYLLPELI<br>GVCHRFNHTRNCTCSERFLVQTLYLHRMSDAEIKLMDRLTSLVNMFPEGFRSSSV***
**NP**	**MDKRVRGSWALGGQSEVDLDYHKILTAGLSVQQGIVRQRIIPVYVVSDLEGICQHIIQAFEAGVDFQDNADSFLLL<br>LCLHHAYQGDHRLFLKSDAVQYLEGHGFRFEVREKENVHRLDELLPNVTGGKNLRRTLAAMPEEETTEANAGQFL<br>SFASLFLPKLVVGEKACLEKVQRQIQVHAEQGLIQYPTSWQSVGHMMVIFRLMRTNFLIKFLLIHQGMHMVAGHD<br>ANDTVISNSVAQARFSGLLIVKTVLDHILQKTDLGVRLHPLARTAKVKNEVSSFKAALGSLAKHGEYAPFARLLNLS<br>GVNNLEHGLYPQLSAIALGVATAHGSTLAGVNVGEQYQQLREAATEAEKQLQQYAETRELDNLGLDEQEKKILMSF<br>HQKKNEISFQQTNAMVTLRKERLAKLTEAITTASKIKVGDRYPDDNDIPFPGPIYDETHPNPSDDNPDDSRDTTIP<br>GGVVDPYDDESNNYPDYEDSAEGTTGDLDLFNLDDDDDDSQPGPPDRGQSKERAARTHGLQDPTLDGAKKVPE<br>LTPGSHQPGNLHITKPGLNTNQPQGNMSSTLQSMTPIQEESEPDDQKDDDDESLTSLDSEGDEDVESVSGENNP<br>TVAPPAPVYKDTGVDTNQQNGPSNAVDGQGSESEALPINPEKRSALEETYYHLLKTQGPFEAINYYHLMSDEPIA<br>FSTESGKEYIFPDSLEEAYPPWLSEKEALEKENRYLVIDGQQFFWPVMSLQDKFLAVLQHD***
**VP24**	**MAKATGRYNLVTPKRELEQGVVFSDLCNFLVTPTVQGWKVYWAGLEFDVNQKGITLLNRLKVNDFAPAWAMTRN<br>LFPHLFKNQQSEVQTPIWALRVILAAGILDQLMDHSLIEPLSGALNLIADWLLTTSTNHFNMRTQRVKDQLSMRM<br>LSLIRSNIINFINKLETLHVVNYKGLLSSVEIGTPSYAIIITRTNMGYLVEVQEPDKSAMDIRHPGPVKFSLLHESTLK<br>PVATPKPSSITSLIMEFNSSLAI***
**VP30**	**MERGRERGRSRNSRADQQNSTGPQFRTRSISRDKTTTDYRSSRSTSQVRVPTVFHKKGTGTLTVPPAPKDICPTL<br>RKGFLCDSNFCKKDHQLESLTDRELLLLIARKTCGSTDSSLNIAAPKDLRLANPTADDFKQDGSPKLTLKLLVETAEF<br>WANQNINEVDDAKLRALLTLSAVLVRKFSKSQLSQLCESHLRRENLGQDQAESVLEVYQRLHSDKGGAFEAALW<br>QQWDRQSLTMFISAFLHVALQLSCESSTVVISGLRLLAPPSVNEGLPPAPGEYTWSEDSTT***
**VP35**	**MQQDRTYRHHGPEVSGWFSEQLMTGKIPLTEVFVDVENKPSPAPITIISKNPKTTRKSDKQVQTDDASSLLTEEVK<br>AAINSVISAVRRQTNAIESLEGRVTTLEASLKPVQDMAKTISSLNRSCAEMVAKYDLLVMTTGRATATAAATEAYWS<br>EHGQAPPGPSLYEDDAIKAKLKDPNGKVPESVKQAYTNLDSTSALNEENFGRPYISAKDLKEIIYDHLPGFGTAFH<br>QLVQVICKIGKDNNILDIIHAEFQASLAEGDSPQCALIQITKRIPAFQDASPPIVHIKSRGDIPKACQKSLRPVPPSPK<br>IDRGWVCIFQFQDGKALGLKI***
**VP40**	**MKRVTVPTAPPAYADIGYPMSMLPIKSSRAVSGIQQKQEVLPGMDTPSNSMRPVADDNIDHTSHTPNGVASAFIL<br>EATVNVISGPKVLMKQIPIWLPLGIADQKTYSFDSTTAAIMLASYTITHFGKANNPLVRVNRLGQGIPDHPLRLLRM<br>GNQAFLQEFVLPPVQLPQYFTFDLTALKLVTQPLPAATWTDETPSNLSGALRPGLSFHPKLRPVLLPGKTGKKGH<br>VSDLTAPDKIQTIVNLMQDFKIVPIDPAKSIIGIEVPELLVHKLTGKKMSQKNGQPIIPVLLPKYIGLDPISPGDLTMVI<br>TPDYDDCHSPASCSYLSEK***

**nuc**	ATTTAGTAAGATGACTTAGCGGACACACAAAAAGATAGAAATGTTTCTTAGACTTTTTGTGTGCGAATAACTATGA<br>GGAAGATTAATCATTTTCCTCAAACTCAAACTAATATTAACATTGAGATTGATCTCATCATTTACCAATTGGAGAC<br>AATTTAACTAGTCAATCCCCCATTTGGGGGCATTCCTAAAGTGTTGCAAAGGTATGTGGGTCGTATTGCTTTGCC<br>TTTTCCTAACCTGGCTCCTCCTACAATTCTAACCTGCTTGATAAGTGTGATTACCTGAGTAATAGACTAATTTCGT<br>CCTGGTAATTAGCATTTTCTAGTAGAACCGATACTATCTCAAGTCCTAAGAGAAGGTGAGAAGAGGGTCCCGAGG<br>TATCCCTCCAGTCCACAAAATCTAGCTAATTTTAGCTGAGTGGACTGATTACTCTCATCACACGCTAACTACTAAG<br>GGTTTACCTGAGAGCCTACAACATGGATAAACGGGTGAGAGGTTCATGGGCCCTGGGAGGGCAATCTGAAGTTG<br>ATCTTGACTACCACAAAATATTAACAGCCGGGCTTTCGGTCCAACAAGGGATTGTGCGACAAAGAATCATCCCGG<br>TATATGTTGTGAGTGATCTTGAGGGTATTTGTCAACATATCATTCAGGCCTTTGAAGCAGGCGTAGATTTCCAAGA<br>TAATGCTGACAGCTTCCTTTTACTTTTATGTTTACATCATGCTTACCAAGGAGATCACAGGCTCTTCCTCAAAAGT<br>GATGCAGTTCAATACTTAGAGGGCCATGGTTTCAGGTTTGAGGTCCGAGAAAAGGAGAATGTGCACCGTCTGGA<br>TGAATTGTTGCCCAATGTCACCGGTGGAAAAAATCTTAGGAGAACATTGGCTGCAATGCCTGAAGAGGAGACAA<br>CAGAAGCTAATGCTGGTCAGTTTTTATCCTTTGCCAGTTTGTTTCTACCCAAACTTGTCGTTGGGGAGAAAGCGT<br>GTCTGGAAAAAGTACAAAGGCAAATTCAGGTCCATGCAGAACAAGGGCTCATTCAATATCCAACTTCCTGGCAAT<br>CAGTTGGACACATGATGGTGATCTTCCGCTTGATGAGAACAAACTTTTTAATCAAGTTCCTACTAATACATCAGG<br>GGATGCACATGGTCGCAGGCCATGATGCGAATGACACAGTAATATCTAATTCTGTTGCCCAAGCAAGGTTCTCTG<br>GTCTTCTGATTGTAAAGACTGTTCTGGACCACATCCTACAAAAAACAGATCTTGGAGTACGACTTCATCCACTGG<br>CCAGGACAGCAAAAGTCAAGAATGAGGTCAGTTCATTCAAGGCAGCTCTTGGCTCACTTGCCAAGCATGGAGAA<br>TATGCTCCATTTGCACGTCTCCTCAATCTTTCTGGAGTCAACAACTTGGAACATGGGCTTTATCCACAACTCTCA<br>GCCATTGCTTTGGGTGTTGCAACTGCCCACGGGAGCACGCTGGCTGGTGTTAATGTAGGGGAGCAATATCAGCA<br>ACTGCGTGAGGCTGCTACTGAAGCTGAAAAGCAACTCCAACAATATGCTGAAACCCGTGAGTTGGACAACCTTG<br>GGCTTGATGAACAGGAAAAGAAGATTCTCATGAGCTTCCACCAGAAGAAGAATGAGATCAGCTTCCAGCAAACT<br>AACGCAATGGTAACCTTGAGGAAAGAGCGGCTGGCCAAGCTGACCGAAGCCATCACGACTGCATCAAAGATCA<br>AGGTTGGAGATCGTTATCCTGATGACAATGATATTCCATTTCCCGGGCCGATCTATGATGAAACCCACCCCAACC<br>CTTCTGATGATAATCCTGATGATTCACGTGATACAACGATCCCAGGTGGTGTTGTTGATCCGTATGATGATGAGA<br>GTAATAATTATCCTGACTACGAGGATTCGGCTGAAGGCACCACAGGAGATCTTGATCTCTTCAATTTGGACGACG<br>ACGATGACGACAGCCAACCAGGACCACCAGACAGGGGGCAGAGCAAGGAGAGAGCAGCTCGGACACATGGCC<br>TCCAAGATCCGACCTTGGACGGAGCGAAAAAGGTGCCGGAGTTGACCCCAGGTTCCCACCAACCAGGCAACCT<br>CCACATCACCAAGCCGGGTTTAAACACCAACCAACCACAAGGCAATATGTCATCTACTCTCCAGAGTATGACCCC<br>TATACAGGAAGAATCAGAGCCCGATGATCAGAAAGATGATGATGATGAGAGTCTCACATCCCTTGACTCTGAAG<br>GTGACGAAGATGTTGAGAGCGTATCAGGGGAGAACAACCCAACTGTAGCTCCACCAGCACCAGTCTACAAGGAT<br>ACTGGAGTAGACACTAATCAGCAAAATGGACCAAGCAATGCTGTAGATGGTCAAGGTTCTGAAAGTGAAGCTCT<br>CCCAATCAACCCCGAAAAGAGATCTGCACTGGAAGAAACATATTATCATCTCCTAAAAACACAGGGTCCATTTGA<br>GGCAATCAATTATTATCACCTAATGAGTGATGAGCCCATTGCTTTTAGCACTGAAAGTGGCAAGGAATATATCTTC<br>CCAGATTCTCTTGAAGAAGCCTACCCGCCTTGGTTGAGTGAGAAGGAGGCCTTAGAGAAAGAAAATCGTTATCT<br>GGTCATTGATGGCCAGCAATTCTTCTGGCCAGTAATGAGCCTACAGGACAAGTTCCTTGCTGTTCTTCAACATGA<br>CTGAGGACCCATGATTAGTAGATTTTGTTTATTCTGAGCTTGATTATAATTGTTTTGATAATTCAAGTATGAGCACC<br>CAACCCGAAATATAAACCCTATTTTAGTTATGAGGAAATTAAATAAATAATCTGTAAGTTGTAGGACTATGAAGAG<br>CTGCTTGTGTCAATTTATCACGGGTTAATACCCATACCGCAAGAATAATTATTTAGTAATTTTAATCAGCTTATAAT<br>ATGTACCAATAGGAAAACATTATAGCATTAAAACATAAAGTATCCTTCGATGAGCTTAGGAGGATAATATCCTGAT<br>GAATTCTATAGAGCTTAGGATTAAGAAAAAATTCATGATGAAGATTAAAACCTTCATCATCCTTTAAAAAGAGAGC<br>TATTCTTTATCTGAATGTCCTTATTAATGTCTAAGAGCTATTATTTTGTACCCTCTTAGCCTAGACACTGCCCAACA<br>TATAAGCCATGCAGCAGGATAGGACTTATAGACATCATGGACCCGAAGTGTCTGGCTGGTTTTCTGAGCAATTAA<br>TGACCGGCAAAATACCGCTAACAGAGGTGTTTGTTGATGTTGAAAACAAACCAAGTCCTGCCCCGATAACCATTA<br>TTAGTAAGAATCCCAAGACAACACGTAAAAGTGATAAGCAAGTCCAAACAGATGATGCCAGTAGCTTATTGACAG<br>AAGAAGTCAAGGCTGCCATAAATTCGGTGATATCAGCTGTGCGTCGGCAAACCAATGCCATTGAATCACTAGAA<br>GGTCGAGTAACAACTCTTGAGGCCAGCTTAAAACCAGTTCAAGACATGGCAAAGACCATATCATCCCTGAATCG<br>CAGCTGTGCCGAAATGGTTGCAAAATACGACCTACTGGTGATGACCACTGGGCGAGCAACTGCCACTGCTGCAG<br>CAACAGAAGCATATTGGAGTGAACATGGACAAGCACCTCCAGGCCCATCATTGTACGAGGATGATGCTATTAAG<br>GCTAAATTGAAAGATCCGAACGGGAAGGTTCCAGAAAGTGTCAAACAGGCCTACACAAATCTAGATAGCACAAG<br>TGCCCTCAATGAGGAAAATTTCGGGCGACCTTACATTTCAGCAAAAGATCTCAAGGAAATCATCTATGACCATCT<br>CCCAGGATTTGGGACAGCTTTTCATCAGTTGGTGCAGGTTATCTGCAAAATTGGTAAGGATAATAATATCCTAGA<br>CATAATTCATGCAGAATTCCAAGCAAGCTTGGCTGAGGGAGACTCCCCCCAGTGTGCATTAATCCAGATAACAAA<br>ACGGATCCCTGCTTTCCAAGATGCCTCTCCTCCAATTGTGCATATCAAGTCTCGAGGAGATATACCCAAAGCCTG<br>TCAGAAAAGCCTCCGGCCGGTCCCACCGTCACCAAAGATCGATAGAGGTTGGGTCTGTATTTTTCAATTCCAAG<br>ACGGGAAGGCCCTTGGGCTAAAAATATGATACAGAAGCAAGGTAAGCTCATTTTGCGATGGCCAAATGATACTTA<br>TGACTGTTCAAAATCAAGTTAGACTAATAGTTTATCATGTCATAAGCTTATAAGTCAGTTTTAAATTTCCCCTCTAT<br>CCTAATCAATTGATAATGCTGTCAATAGGGAAATTCCCCTGTATTGTAATAAGACCTCATTAACATATTTCCCCTGC<br>TTAGTACTATGCAGAAACCCCCGAGCAAATTAAAATTGATGAAGATTAAGAAAAAGAGGGATTTTCTCAGGAAAA<br>ATCTTTTTTCTTACCTTCATCTCATTTAAACAAATTTAGGACTCAGGAAAAATGAAAAGGGTCACTGTGCCGACTG<br>CACCACCTGCCTATGCTGACATTGGCTATCCTATGAGCATGCTTCCCATCAAGTCAAGCAGGGCTGTGAGTGGAA<br>TTCAACAGAAACAAGAGGTCCTTCCTGGAATGGATACACCATCAAATTCTATGAGACCTGTTGCTGATGATAACA<br>TTGATCATACAAGTCATACCCCGAACGGAGTGGCCTCAGCATTCATCTTGGAGGCAACTGTCAATGTGATCTCGG<br>GGCCCAAAGTCCTCATGAAACAAATCCCTATTTGGTTGCCACTCGGAATTGCTGACCAAAAAACGTACAGTTTTG<br>ACTCAACAACAGCAGCAATTATGCTCGCATCTTATACGATCACCCATTTTGGAAAGGCCAACAACCCCCTCGTTA<br>GAGTGAATCGACTTGGTCAGGGAATACCGGATCACCCACTCAGATTGCTCAGGATGGGGAACCAGGCTTTCCTT<br>CAAGAGTTTGTGCTACCACCAGTTCAACTGCCGCAATATTTCACTTTTGATCTGACTGCACTCAAATTAGTGACA<br>CAGCCTCTCCCTGCTGCAACATGGACAGATGAGACTCCGAGCAACCTTTCAGGAGCACTTCGTCCCGGGCTTTC<br>ATTTCACCCAAAGCTGAGACCCGTTCTACTTCCAGGCAAAACGGGAAAGAAAGGGCATGTTTCTGATCTGACTG<br>CCCCAGACAAAATTCAGACAATTGTGAACCTGATGCAAGATTTCAAAATCGTGCCAATTGATCCAGCTAAGAGTA<br>TCATTGGGATCGAGGTTCCAGAATTGCTGGTCCACAAGCTCACCGGGAAGAAAATGAGTCAGAAGAATGGACAG<br>CCTATAATTCCTGTCTTACTTCCAAAATACATTGGGCTAGATCCAATCTCACCTGGAGACCTGACTATGGTCATAA

CACCAGATTATGATGATTGTCATTCACCTGCCAGTTGCTCTTATCTCAGTGAAAAGTGATTCTCACAAAGTGAGA
GAAACACCTCCAGTAAAGAAATCAAATCTTATCTATAGCAACTCAATCGACTTTTAGGAAGCTAGCAGTACATATA
CTATGGGACAACTCAACCCTCTTGTTAAAATGTACTAATCGGTTCAAGGAACTCTCACTGATCAAGCCTGAATCC
AAGATAGAACCAGCCCAAAGGGCCCCCCAGAGTCTCCCACAAGCTTAGCCAATCAATTAACATGCATAAGCGA
TCCATACTTCGCCCAATCAGTGTCCGATGTTCACCCCTTCAAGCCTCCTTCCTAGCAAATTGACCTAGCTGTACC
AAGAGATTCCCTCAGCCTCCTTCTCAAATAACCTGATCCTTGAGGGTTATACCTTCACCACTTTATGCTCATTTCA
CCCAAACATAAAATGAAATGTCTTAACATGATTGCACCATTAAGAAAAACAAATCTGATGAAGATTAAGCCTGATT
AAGGCCCAACCTTCATCTTTTTACCATAATCTTGTTCTCAGTACCATTTGATAAGGGTACACTTGCCAAAACGCCC
CCATCCTAAGGGTCTCGCAATGGGGAGTCTTAGCCTACTCCAATTGCCCAGGGACAAATTTCGGAAAAGCTCTTT
CTTTGTTTGGGTCATCATCTTATTCCAAAAGGCCTTTTCCATGCCTTTGGGTGTTGTGACTAACAGCACTTTAGAA
GTAACAGAGATTGACCAGCTAGTCTGCAAGGATCATCTTGCATCTACTGACCAGCTGAAATCAGTTGGTCTCAAC
CTCGAGGGGAGCGGAGTATCTACTGATATCCCATCTGCAACAAAGCGTTGGGGCTTCAGATCTGGTGTTCCTCC
CAAGGTGGTCAGCTATGAAGCGGGAGAATGGGCTGAAAATTGCTACAATCTTGAAATAAAGAAGCCGGACGGGA
GCGAATGCTTACCCCCACCGCCAGATGGTGTCAGAGGCTTTCCAAGGTGCCGCTATGTTCACAAAGCCCAAGGA
ACCGGGCCCTGCCCAGGTGACTACGCCTTTCATAAGGATGGAGCTTTCTTCCTCTATGACAGGCTGGCTTCAAC
TGTAATTTACAGAGGAGTCAATTTTGCTGAGGGGGTAATTGCATTCTTGATATTGGCTAAACCAAAAGAAACGTT
CCTTCAGTCACCCCCCATTCGAGAGGCAGTAAACTACACTGAAAATACATCAAGTTATTATGCCACATCCTACTTG
GAGTATGAAATCGAAAATTTTGGTGCTCAACACTCCACGACCCTTTTCAAAATTGACAATAATACTTTTGTTCGTC
TGGACAGGCCCCACACGCCTCAGTTCCTTTTCCAGCTGAATGATACCATTCACCTTCACCAACAGTTGAGTAATA
CAACTGGGAGACTAATTTGGACACTAGATGCTAATATCAATGCTGATATTGGTGAATGGGCTTTTTGGGAAAATA
AAAAAATCTCTCCGAACAACTACGTGGAGAAGAGCTGTCTTTCGAAGCTTTATCGCTCAACGAGACAGAAGACG
ATGATGCGGCATCGTCGAGAATTACAAAGGGAAGAATCTCCGACCGGGCCACCAGGAAGTATTCGGACCTGGTT
CCAAAGAATTCCCCTGGGATGGTTCCATTGCACATACCAGAAGGGGAAACAACATTGCCGTCTCAGAATTCGAC
AGAAGGTCGAAGAGTAGGTGTGAACACTCAGGAGACCATCACAGAGACAGCTGCAACAATTATAGGCACTAACG
GCAACCATATGCAGATCTCCACCATCGGGATAAGACCGAGCTCCAACCAAATCCCGAGTTCCTCACCGACCACG
GCACCAAGCCCTGAGGCTCAGACCCCCACAACCCACACATCAGGTCCATCAGTGATGGCCACCGAGGAACCAA
CAACACCACCGGGAAGCTCCCCCGGCCCAACAACAGAAGCACCCACTCTCACCACCCCAGAAAATATAACAACA
GCGGTTAAAACTGTCCTGCCACAGGAGTCCACAAGCAACGGTCTAATAACTTCAACAGTAACAGGAATTCTTGG
GAGTCTTGGGCTTCGAAAACGCAGCAGAAGACAAACTAACACCAAAGCCACGGGTAAGTGCAATCCCAACTTAC
ACTACTGGACTGCACAAGAACAACATAATGCTGCTGGAATTGCCTGGATCCCGTACTTTGGACCGGGTGCGGAA
GGCATATACACTGAAGGCCTGATGCATAACCAAAATGCCTTAGTCTGTGGACTTAGGCAACTTGCAAATGAAACA
ACTCAAGCTCTGCAGCTTTTCTTAAGAGCCACAACGGAGCTGCGGACATATACCATACTCAATAGGAAGGCCATA
GATTTCCTTCTGCGACGATGGGGCGGGACATGCAGGATCCTGGGACCAGATTGTTGCATTGAGCCACATGATTG
GACAAAAAACATCACTGATAAAATCAACCAAATCATCCATGATTTCATCGACAACCCCTTACCTAATCAGGATAAT
GATGATAATTGGTGGACGGGCTGGAGACAGTGGATCCCTGCAGGAATAGGCATTACTGGAATTATTATTGCAATT
ATTGCTCTTCTTTGCGTTTGCAAGCTGCTTTGCTGAATATCAATTTGAATCATCAATTTAAGCTTGATACATTTCTA
GCATTTTAAATTATAAACCGATACTGATACTTGAAAATCAGGCTAATGCCAAGTTCTGTGCAAAACTTGAAAGTAG
GTTTACAAAAATCCTTTGAACTGGAATGCTTTGATACTCTTTCTCAATACTATATAAGTTCCTTCCCAAGAATAATA
TTGATGAAGATTAAGAAAAAGTGACATTGTGCCCACTTTTGTAATCTTCATCCACCTACACATTCATATTCAGGAA
TCTTTGAATTAACCCTCACACTTGCTTAGGAAAGAGCCTATCCTCTACAAGAATCCCGAGGCGGCAATTCAGTTA
ATTTCATATAAAGATAATATCCATTTCCAAGACCACAGATAACTATATTATTAATCTTTACCACAAATATGGAGAGG
GGTCGTGAGCGCGGGAGATCAAGGAATTCACGTGCCGACCAGCAAAATTCAACAGGTCCTCAATTTAGGACAAG
ATCCATTTCCCGGGATAAGACAACAACAGACTACCGTAGTAGTCGAAGTACTTCGCAAGTTAGAGTCCCTACGGT
TTTCCATAAGAAAGGTACTGGGACCCTTACTGTCCCTCCAGCACCTAAGGATATTTGTCCTACTCTCAGAAAAGG
ATTTCTATGTGATAGTAATTTCTGTAAAAAGGACCATCAACTTGAAAGCCTAACCGACCGGGAGCTCCTACTTCTT
ATAGCACGGAAGACCTGTGGATCAACTGATTCATCGCTTAATATAGCTGCTCCTAAAGACCTAAGACTAGCAAAT
CCTACGGCTGATGACTTCAAGCAAGACGGCAGTCCAAAATTAACCCTAAAATTACTAGTCGAGACTGCTGAGTTT
TGGGCCAATCAGAATATTAATGAAGTAGATGATGCAAAACTCCGTGCTCTCTTGACGTTGAGTGCTGTCTTAGTG
CGGAAATTCTCTAAGTCACAGCTTAGTCAATTATGTGAGAGTCATCTTAGGAGGGAAAACTTAGGACAAGACCAA
GCTGAATCAGTTCTCGAGGTTTATCAACGTTTACATAGTGATAAAGGAGGTGCTTTTGAGGCAGCACTATGGCAA
CAGTGGGATAGACAATCATTAACTATGTTTATATCTGCTTTCCTCCATGTAGCATTGCAACTTTCCTGTGAGAGCT
CCACTGTAGTGATATCAGGCCTACGCTTACTTGCCCCCCCAAGCGTTAATGAAGGGCTCCCTCCTGCACCAGGG
GAATATACTTGGTCAGAAGATAGTACAACTTAGCCTGTAGGGAGGACAAGTAAAACAAGATGCCCTTATCCTCTA
TAGATGGTATTTTTAAAGAGGAGGACAGGATAGGAATAAAGATAATGACTAAAGCCAATATAAAGATACGAACAC
AAGTAGAAATTAAAATAGAAATCAAAACAATCTCCCCTTATTCAATATGAAATATAATAGTGAGTATTTGTTTCATA
ATGTCAATCATTTATTGTTAAAAATAAACAAAGTCAGTAAGAGTGTTAGGATCGTTATATTGCAAGGATCCTCCCT
AGAAGCGTTGAATCATCTCAAGTAGCCTAGAACAAGAACAGCAGAACATTAAATTGAAATAGATAATAAGGATAT
CGCTTGTTTTTAAGATAGTTTTAGGAAGTTTAAAATTAAGAAAAAGAACTCATGGACACACTCTAGCATTGAGGA
TGGGGTTCCCTTGATGATAGTATAGTCTTAGGTATAGGGTAGTCCTACACGTCCTATATTATACAGTCTAAACTTG
TAAAATTAAACTACAAGAACATGATGAAAATTAATGAGAAGGTTCCAAGATTGACTTCAATCCAAACACCTTGCT
CTGCCAATTTTCATCTCCTTAAGATATATGATTCTGTTCCTGCGAGATAAGGTTATCAAATAGGGTGTGTATCTCTT
TTACATATTTGGGCTCCCACTAGGCTAGGGTTTATAGTTAAGGAAGACTCATCACATTTTTTATTGAACTAGTCTA
CTCGCAGAATCCCACCGGGAATAGAAATTAGAACATTTGTGATACTTTGACTATAGGAAATAATTTTCAACACTAC
CTGAGATCAGGTTATTCTTCCAACTTATTCTGCAAGTAATTGTTTAGCATCATAACAACAACGTTATAATTTAAGAA
TCAAGTCTTGTAACAGAAATAAAGATAACAGAAAGAACCTTTACTATACGGGTCCATTAATTTTATAGGAGAAGCT
CCTTTTACAAGCCTAAGATTCCATTAGAGATAACCAGAATGGCTAAAGCCACAGGCCGGTACAACTTGGTAACAC
CAAAACGGGAGCTAGAGCAAGGAGTTGTGTTTAGCGACCTATGCAACTTCCTAGTGACTCCAACTGTGCAAGGA
TGGAAGGTTTACTGGGCTGGACTTGAGTTTGACGTCAACCAAAAGGGTATTACCCTGTTAAATCGTCTTAAAGTG
AATGATTTTGCTCCTGCATGGGCGATGACCCGGAACCTCTTCCCACACTTGTTCAAAAACCAACAGTCTGAAGTC
CAAACTCCCATTTGGGCTTTGAGGGTAATTCTTGCCGCCGGGATTCTTGACCAATTAATGGATCATTCCCTCATT
GAGCCGCTATCAGGGGCCCTGAACCTAATTGCTGATTGGTTACTAACAACATCTACTAATCACTTCAACATGAGA
ACTCAACGAGTAAAGGACCAACTGAGCATGAGGATGTTATCTCTTATAAGGTCAAATATTATTAACTTTATAAATA

AGCTCGAGACTCTTCATGTCGTTAATTACAAGGGACTTCTAAGCAGTGTTGAGATAGGAACACCAAGCTATGCAA
TCATTATTACCAGGACTAATATGGGTTATCTTGTCGAGGTTCAGGAACCAGATAAATCTGCGATGGATATACGACA
CCCTGGTCCTGTCAAATTCTCCCTACTACATGAATCGACACTTAAACCTGTTGCCACTCCTAAACCGTCAAGCATT
ACTTCATTGATCATGGAGTTCAACAGTTCTCTGGCAATTTAATTGCCGTAATAAAAATTGTACGATAGGGCTAACA
TTGATTCCATAATCCATTGTAGGACAGAATCATTTTCCTGTATGATCTTAGTTTAATCTCTCTTTATACAATGATGA
ATAAGGAGCTTGTTTAAAATGTTACAAAAGTATACTGTTTGAACCCCTAGTATCCCTGTAAATATCCTCATTCAATT
TTTTGTTTTTACATGTGTAGTCACCTGTATAGCATGACCCTAGTCATGCCTCTAATTAATACTTAATCTAACAGTTA
ATATAATGTATAACTTTCCATGTTCAAAGAGTAGTCAAAACAATGTGAGATCCAGTTTCACTCACAGCATCTATTC
ACTATTTACAGTATGATGAGCCCAAATTAACACAGTAGAGGTCTAGATTTATTAATAGAACGAGGAAGATTAAGAA
AAAGTCCATAATGCTGGGGAGGCAATCCCTGCCACCATAGGACTTTTTCAATTCCTCTATTTTATGATGGCTACCC
AACATACACAATATCCTGATGCAAGATTGTCTTCCCCAATTGTCTTAGACCAATGTGACCTAGTGACAAGAGCAT
GTGGACTTTACTCTGAGTATTCGCTGAACCCTAAACTAAGGACATGCCGTTTACCGAAACATATCTATAGATTAAA
ATATGACACTATTGTTTTACGATTTATTAGTGATGTCCCTGTAGCTACAATCCCAATAGACTACATTGCTCCGATGT
TAATAAATGTTCTGGCAGATAGTAAAAATGTACCATTGGAACCTCCCTGCTTGAGTTTCTTGGATGAAATAGTCAA
TTATACCGTGCAGGATGCAGCATTCCTTAATTATTACATGAATCAGATTAAAACACAGGAAGGAGTAATTACAGAT
CAATTAAAACAGAACATTCGTAGGGTCATTCACAAAAACAGATATCTATCTGCTCTATTCTTCTGGCATGATCTTG
CCATCCTCACCCGTCGAGGGAGAATGAACCGAGGAAATGTGCGCTCCACTTGGTTTGTAACGAATGAGGTTGTT
GACATTCTAGGATATGGTGATTATATCTTCTGGAAGATCCCTATTGCTCTATTACCAATGAACACAGCTAATGTTC
CACATGCATCAACTGACTGGTACCAACCTAATATCTTCAAGGAGGCTATTCAAGGACACACACATATTATTTCAGT
CTCTACAGCCGAGGTCCTTATTATGTGTAAGGATCTTGTCACAAGTCGTTTTAATACCCTTCTGATTGCTGAGTTA
GCCAGGTTGGAAGATCCAGTGTCTGCTGATTATCCACTAGTAGATAATATTCAATCTCTGTATAACGCAGGAGACT
ACCTGTTGTCCATATTGGGATCAGAGGGGTACAAAATAATCAAATATCTCGAACCTCTGTGTTTGGCTAAGATTC
AACTATGTTCCCAATATACAGAACGAAAAGGGCGGTTTTTAACCCAGATGCATCTTGCAGTTATTCAAACATTGC
GTGAACTCCTCCTTAATAGAGGGTTGAAAAAATCACAATTGTCTAAAATCCGCGAGTTTCACCAACTGTTGCTCA
GACTCCGATCCACACCACAACAATTATGTGAATTATTTTCAATCCAAAAACACTGGGGCCACCCAGTTCTGCATA
GTGAAAAGGCCATCCAAAAGGTTAAAAATCATGCAACAGTTCTAAAGGCATTGCGGCCGATTATCATCTTTGAAA
CGTATTGTGTATTCAAGTATAGTGTTGCAAAACATTTCTTTGATAGTCAAGGCACTTGGTACAGTGTGATATCAGA
CCGATGTTTAACGCCGGGATTGAATTCCTACATTAGGCGAAATCAATTCCCTCCACTTCCAATGATCAAAGATCTT
TTATGGGAATTTTACCATTTGGATCATCCTCCATTATTCTCCACGAAGATCATTAGTGACCTCAGCATTTTCATTAA
AGACCGCGCAACAGCAGTTGAACAAACCTGTTGGGATGCAGTTTTTGAGCCTAACGTTTTGGGCTACAGTCCAC
CTTATCGATTCAATACCAAACGTGTACCTGAACAATTCCTGGAGCAAGAGGATTTTTCTATTGAGAGTGTCTTACA
ATACGCCCAAGAACTTAGGTACTTATTGCCCCAGAATCGAAATTTTTCTTTTTCATTGAAGGAAAAAGAATTAAAT
GTTGGTAGGACATTTGGAAAATTGCCTTATTTAACCAGGAATGTCCAAACCCTCTGCGAAGCATTACTTGCAGAT
GGTTTGGCTAAAGCCTTTCCAAGCAATATGATGGTTGTCACAGAGAGGGAACAAAAGGAGAGCCTCCTTCACCA
AGCATCCTGGCACCATACAAGTGATGATTTCGGAGAGCATGCCACAGTTCGTGGAAGTAGTTTTGTCACAGACC
TGGAAAAATACAATCTGGCCTTCAGGTATGAATTCACAGCTCCCTTCATCAACTATTGCAACCAATGCTATGGGG
TTCGCAATGTCTTTGATTGGATGCACTTCCTAATTCCGCAATGTTACATGCATGTTAGTGATTATTATAACCCACC
ACATAATGTAACCTTAGAGAATAGGGAATATCCCCCCGAAGGACCAAGTGCTTATAGAGGCCACCTTGGCGGTAT
TGAGGGGCTTCAACAAAAGTTATGGACTAGTATCTCATGTGCTCAAATCTCATTGGTAGAGATCAAGACCGGGTT
CAAATTGCGATCAGCAGTCATGGGGGATAATCAATGTATTACAGTATTATCAGTCTTTCCACTAGAATCTAGTCCG
AATGAGCAGGAGAGATGCGCAGAAGACAATGCAGCCAGAGTGGCTGCTAGCTTGGCCAAAGTCACAAGTGCCT
GTGGGATATTCCTCAAGCCTGATGAGACTTTTGTACACTCAGGCTTTATCTATTTTGGCAAAAAGCAATACTTGAA
CGGAATTCAATTACCTCAATCACTCAAGACAGCAGCTAGGATGGCCCCTCTCTCAGATGCAATTTTTGATGACTT
GCAAGGTACACTTGCCAGTATAGGAACTGCCTTTGAGCGATCAATCTCCGAAACTAGACATATTTTACCATGTCG
TGTTGCAGCTGCCTTTCATACATATTTCTCTGTTCGGATCTTACAACATCATCACCTTGGTTTCCATAAGGGTTCA
GACCTTGGACAATTGGCAATCAATAAACCTCTTGATTTCGGAACCATTGCACTATCCTTAGCAGTTCCTCAGGTAT
TGGGTGGATTATCCTTCCTAAATCCAGAAAAGTGCCTTTATCGCAACTTGGGTGATCCTGTAACTTCAGGCCTAT
TTCAGTTGAAGCATTATCTGTCAATGGTGGGTATGAGTGATATCTTTCATGCACTTGTTGCAAAAAGCCCAGGGA
ATTGTAGCGCAATTGACTTTGTTCTAAACCCAGGCGGGTTAAATGTCCCTGGATCACAGGATTTAACATCTTTCC
TTCGTCAGATTGTCAGAAGGAGTATCACACTTTCGGCAAGAAACAAGTTAATCAACACGTTATTTCACGCTTCTG
CAGATCTTGAAGACGAGTTAGTATGTAAATGGTTACTTTCTTCAACGCCCGTGATGAGCCGTTTCGCAGCCGATA
TTTTCTCACGAACACCAAGCGGGAAAAGATTACAAATCTTGGGATACCTCGAGGGAACCAGAACTTTATTAGCAT
CCAAAATGATAAGCAATAATGCAGAGACACCAATCTTGGAGAGGCTCAGAAAAATAACGCTTCAAAGATGGAATC
TATGGTTTAGTTACCTAGACCATTGTGACCCAGCTTTAATGGAAGCAATTCAACCAATTAAGTGTACTGTTGATAT
TGCTCAAATTCTTAGAGAATACTCCTGGGCTCATATTCTTGATGGTAGACAGTTAATAGGGGCAACACTGCCATG
TATACCTGAGCAGTTCCAAACCACATGGTTAAAACCTTACGAGCAATGTGTGGAATGTTCATCCACAAACAATTC
TAGTCCATATGTATCAGTTGCATTAAAAAGGAACGTGGTTAGTGCTTGGCCTGATGCGTCTAGATTGGGGTGGAC
GATTGGTGATGGGATTCCCTACATAGGCTCAAGAACTGAGGACAAGATAGGTCAGCCCGCTATTAAGCCGAGGT
GCCCATCAGCTGCATTAAGAGAAGCTATTGAATTGACCTCTAGGTTGACCTGGGTCACTCAAGGTAGTGCAAAC
AGCGATCAGTTAATTCGCCCTTTTCTTGAGGCAAGAGTAAACTTGAGTGTACAAGAGATTCTTCAAATGACCCCC
TCACATTACTCCGGTAATATTGTGCATCGGTATAATGATCAGTATAGCCCTCACTCCTTTATGGCTAACCGCATGA
GTAACACAGCAACGCGCTTGATGGTATCTACCAACACACTAGGAGAGTTTTCCGGAGGGGGTCAGGCTGCACGT
GATAGCAACATTATATTTCAAAATGTGATTAACTTTGCAGTGGCCTTGTATGACATTAGGTTTCGGAACACTTGTA
CATCTTCTATTCAATATCACAGGGCCCATATTCACCTGACGAATTGTTGTACGAGGGAAGTACCGGCCCAATACTT
AACATACACAACCACGCTAAATCTAGATTTGAGTAAGTACCGTAATAATGAACTGATTTATGATTCAGAGCCACTA
AGAGGAGGTCTCAACTGCAACTTATCGATTGACAGTCCTTTGATGAAGGGCCCACGTTTAAATATTATTGAGGAT
GACTTAATACGGTTGCCACATTTATCCGGCTGGGAATTAGCAAAAACAGTCTTGCAATCAATAATCTCTGATAGTA
GCAATTCATCAACAGATCCCATTAGCAGCGGTGAAACAAGATCCTTCACAACCCACTTCTTAACGTATCCCAAAA
TAGGGCTCCTATACAGTTTTGGAGCCCTCATAAGTTTTTATTTGGGTAATACTATTCTGTGCACGAAAAAGATCGG
ACTCACAGAATTTCTATACTATCTCCAGAATCAGATCCACAACTTATCACACAGATCCCTTCGAATCTTCAAACCG
ACATTTAGACACTCAAGTGTCATGTCCAGGTTGATGGATATAGACCCCAACTTCTCAATATATATTGGTGGGACTG
CAGGTGACCGTGGATTATCGGACGCTGCAAGATTATTTCTCCGAATTGCAATTTCAACTTTCTTGAGCTTTGTTG

AGGAGTGGGTTATCTTTAGGAAGGCAAACATCCCACTATGGGTTATCTATCCTCTCGAAGGCCAACGCCCTGATC
CTCCTGGCGAATTTTTGAACCGAGTAAAATCTCTAATTATTGGGACTGAAGATGATAAAAATAAAGGTTCTATACT
TTCAAGATCTGGAGAGAAATGCTCTTCAAATTTAGTTTATAATTGCAAGAGTACAGCAAGCAATTTTTTCCATGCA
TCATTGGCTTACTGGAGAGGTCGACATAGACCTAAGAAGACCATAGGTGCAACTAACGCGACAACAGCTCCACA
TATCATTTTGCCACTAGGAAATTCTGATCGACCGCCTGGCCTAGACCTTAATAGGAACAATGATACTTTCATTCCT
ACCAGAATTAAACAGATAGTCCAAGGAGATTCTAGAAACGACAGAACGACCACCACGAGATTTCCACCCAAAAG
TAGGTCCACTCCAACATCAGCAACCGAGCCTCCTACAAAAATGTATGAGGGTTCGACAACCCACCAAGGGAAAT
TAACAGATACACATTTGGATGAGGATCACAATGCCAAAGAGTTCCCATCCAATCCGCATCGTTTAGTAGTACCATT
CTTTAAATTAACAAAAGATGGGGAATACAGTATCGAACCTTCTCCTGAAGAAAGCCGCAGTAATATAAAAGGGTT
ACTTCAACATTTAAGAACCATGGTTGATACTACCATATATTGTCGCTTCACTGGAATTGTTTCATCAATGCATTATA
AGTTAGATGAAGTACTATGGGAATATGATAAATTTGAATCAGCTGTAACCCTAGCAGAAGGGGAGGGTTCAGGTG
CCTTACTACTGATCCAAAAATACGGCGTTAAGAAGTTATTTTTGAATACACTTGCTACTGAACATAGTATTGAGAG
TGAAGTGATATCAGGTTACACCACTCCAAGGATGCTACTCTCAATTATGCCTAAAACACATCGTGGTGAGCTAGA
GGTCATATTAAATAACTCAGCTAGTCAAATAACTGATATTACACACCGAGATTGGTTTTCAAATCAAAGAAATAGG
ATTCCAACTGATGCTGATATTATTACCATGGATGCTGAAACTACAGAAAACTTAGATCGTTCCAGATTATATGAAG
CAGTATATACGATTATTTGTAATCATATCAATCCTAAAACTTTGAAAGTGGTCATCTTAAAAGTCTTCCTCAGCGAT
TTGGATGGGATGTGCTGGATTAACAATTATCTTGCTCCTATGTTTGGATCAGGATATTTAATCAAACCTATAACAT
CAAGTGCAAAGTCAAGTGAGTGGTATTTATGCTTATCTAATCTACTTTCAACCTTGAGAACTACTCAGCATCAAAC
CCAGGCAAACTGTCTCCATGTCGTACAATGTGCTCTTCAACAGCAAGTACAAAGAGGGTCATATTGGCTAAGTCA
TCTTACTAAATACACCACAAGTAGATTGCACAATAGTTATATTGCATTTGGTTTTCCTTCATTAGAGAAGGTCCTAT
ATCATAGGTATAACCTTGTTGATTCGAGAAACGGACCATTAGTTTCTATAACGAGACACCTTGCCCTCCTCCAAAC
TGAGATCAGGGAGTTGGTAACTGATTATAATCAGCTGCGACAAAGTCGAACCCAGACTTATCATTTCATAAAAAC
ATCCAAGGGACGGATAACTAAACTAGTGAATGATTATCTAAGATTTGAGTTGGTTATACGGGCTCTTAAAAATAAT
TCTACATGGCACCATGAGTTATACTTGCTACCAGAACTTATAGGTGTTTGCCATCGATTTAATCATACACGTAACT
GTACATGCAGCGAAAGGTTCCTGGTTCAAACTTTATATCTACACCGAATGAGTGATGCTGAGATAAAACTTATGG
ACCGGCTCACCAGCCTAGTCAATATGTTTCCTGAAGGTTTCAGGTCTAGTTCAGTCTAATTCTAACTGCACCAAA
GGCTCTAGAAATATTTTAAATAACCAGGTGTATATCAAAGTCAATACAAGTGTAAAAACAATATGCAAGGGACCAC
ATTGAGGATCAGTTTATTGACTCTTCCAATACACAGAGTTGGAAGCACCGATTCAAGGTTTCTAAGACGCTCTAT
CAATTATGTTGATAATGTAAATAATAGCTTTTCCTGCCTATTATGACTTAAATAAACATATCTATAACGACCATCACA
GCTAAGTCGTTGCCCTAGTTCATATATTAAATTAAAATTTAGAAGCTAGGTTAACTCTAATTACATAAGTATTAAGA
AAAAATTACTAAGACTAATACTCTCATGCCAAGAACTAGTAATGTGTTTCACATGACAGATTATTTCTAACACTAA
ATTGCAATTTCAATTTTAAAGCTAAGTTTAACACCTATACAGCCAAAATATTTCATAGGGCCGATGGGAATAACAT
AAGAGGAACATGATCAATGAACCCTTTATTCCAACTAGGCAGTTGATTGATAATCTACAAATTCCATAAGATGTTC
TTACGATATTCTTTTGTTTTTAATCTCAATGTCAATGATTTAATAAGTAATAATAAAAAAATCACATTAAAGATGCA
GGAAGATCTTGACCTCGCCAGGAAAATTAAGCGCACACAAATAAATTAAAAAATCTGTATTTTCTCTTTTTTGAGT
GCACA
